# Ancient Hulled Wheat: An Antioxidant-Rich Crop for Boron-Contaminated Soils

**DOI:** 10.1021/acsomega.4c11314

**Published:** 2025-04-08

**Authors:** Ridvan Temizgul

**Affiliations:** Department of Biology, Faculty of Sciences, Erciyes University, Kayseri 38039, Türkiye

## Abstract

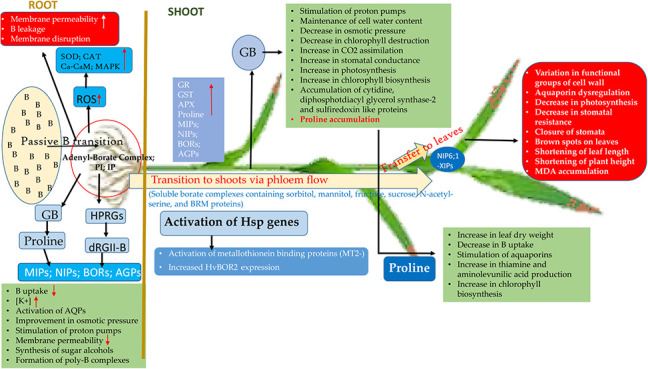

This study investigated the boron (B) tolerance of four ancient hulled wheat species, examining their morphological, physiological, and antioxidant responses to varying B concentrations and the mitigating effects of exogenous glycine betaine (GB). Results revealed that B initially promoted root and shoot biomass, but higher concentrations induced growth inhibition, mitigated by GB application. B exposure increased total protein content and antioxidant enzyme activities at lower concentrations but decreased them at higher concentrations, indicating oxidative stress. Exogenous GB enhanced antioxidant enzyme activities and proline accumulation, alleviating oxidative damage. These findings suggest varying B tolerance among ancient hulled wheat varieties. GB effectively mitigated B-induced stress by bolstering antioxidant defenses and promoting osmotic adjustment. This highlights the potential of ancient hulled wheat as a genetic resource for developing B-tolerant wheat cultivars.

## 1 Introduction

Boron (B), while an essential micronutrient for plant growth and development, can become toxic at elevated levels. Excessive B disrupts physiological processes, leading to chlorosis, necrosis, and ultimately, reduced yield or plant death.^1−4^ Plant species exhibit varying sensitivities to B, with tolerant species showing resilience to both deficiency and excess, while sensitive species are more susceptible to fluctuations in B concentration. The primary source of B is ocean evaporation (65–85%), with additional contributions from the weathering of sedimentary rocks and B-related mining activities.^5,6^ Borax (Na_2_B4O_7_·10H_2_O) and boric acid [B(OH)_3_] are commonly used water-soluble B fertilizers.^7^ Unlike other pollutants, human activities have a minimal impact on environmental B release compared with natural sources. Arid and semiarid regions are particularly vulnerable to B toxicity due to capillary action and evaporation of boron-rich groundwater. Under such conditions, B concentrations can exceed toxic thresholds, impacting agricultural productivity and leading to crop yield losses.^8^ Soils with water-soluble B concentrations exceeding 5 mg L^–1^ are considered B-toxic.^9^

Boron, primarily present as boric acid in soil solutions, is highly soluble and susceptible to leaching. Consequently, boron toxicity is prevalent in alkaline and saline soils of arid and semiarid regions characterized by low precipitation and minimal leaching. In these environments, excess boron accumulates in topsoil layers due to the evaporation of boron-rich groundwater.^8,10,11^ The detrimental impacts of boron excess on agricultural productivity and ecosystems have been extensively documented.^12,13^ Countries such as Australia, Egypt, Iraq, Syria, Türkiye, and California are particularly affected by boron toxicity in agricultural areas.^14^ Conversely, many nations, including Japan, China, and the USA, experience boron deficiency in their soils and require boron supplementation through fertilization.^15^ Among the naturally occurring and commercially significant borate compounds, tincal, colemanite, kernite, ulexite, and boric acid are the most prominent. The global supply of these compounds is primarily concentrated in Türkiye and the United States, which collectively account for approximately 90% of the world’s borate production.^16^

Boron, though essential for plant growth, is required in minute quantities. Optimal levels for plant health range from 0.5 to 2.0 mg L^–1^. Soils deficient in boron, typically below 0.5 mg L^–1^, can lead to visible deficiency symptoms. Conversely, excessive boron, exceeding 2.0 mg L^–1^, can induce toxicity, resulting in reduced yield and product quality. Characteristic symptoms of boron toxicity include marginal chlorosis and necrosis of mature leaves, progressing from leaf tips toward the interior. This foliar damage compromises photosynthetic capacity, negatively impacting plant productivity.^17^ As an uncharged molecule, boric acid readily permeates lipid bilayers, with uptake directly proportional to the concentration gradient.^8^ Boron transport to aerial plant parts is primarily via xylem loading and transpiration. The final accumulation sites for boron are often the tips and margins of mature leaves.^18^

Türkiye is a significant global producer of natural borax fertilizers.^19^ The country boasts over 50% of the world’s boron reserves,^20^ making boron a strategic element in technological advancements.^21^ With proven and probable reserves of 375 and 483 million tons, respectively, Türkiye holds 72.2% of the world’s total boron reserves.^22^ Aqueous waste generated from mining operations typically contains 14–18% B_2_O_3_, which accumulates in collection ponds.^21^ These pond water concentrations often exceed World Health Organization (WHO) limits.^23^ Annual boron mining waste production in Türkiye amounts to approximately 60,000 tons.^24^

Numerous studies have demonstrated that excessive boron levels can lead to the overproduction of reactive oxygen species (ROS) in various plant species.^25,26^ Oxidative stress, induced by ROS, can result in chromosomal abnormalities and DNA damage.^27^ Boron’s genotoxic potential is manifested either through ROS generation or via other toxicity mechanisms.^28^ Plants possess an antioxidant defense system within cellular organelles, comprising enzymatic and nonenzymatic antioxidants that effectively scavenge ROS. However, when ROS production surpasses the scavenging capacity of this system, oxidative damage ensues.

Nonenzymatic antioxidants, including ascorbate (AsA), glutathione (GSH), carotenoids, tocopherols, and flavonoids, are key components of this defense system. Enzymatic antioxidants, such as ascorbate peroxidase (APX), monodehydroascorbate reductase (MDHAR), dehydroascorbate reductase (DHAR), glutathione reductase (GR), superoxide dismutase (SOD), catalase (CAT), glutathione peroxidase (GPX), glutathione S-transferase (GST), and peroxiredoxin (PRX), play crucial roles in ROS detoxification.^29^ Oxidative stress is a primary consequence of ionic and osmotic stress induced by boron exposure.^30^ This stress disrupts photosynthesis and leads to the excessive production of ROS, including singlet oxygen (^1^O_2_), superoxide (O_2_^–^), hydrogen peroxide (H_2_O_2_), and hydroxyl radicals (OH•).^31^ To mitigate this stress, plants synthesize compatible osmolytes like proline and glycine betaine (GB), as well as nonenzymatic antioxidants such as AsA and GSH, and upregulate enzymatic antioxidants to detoxify ROS to manageable levels.^32^ Exogenous application of osmoprotectants, such as proline and GB, has been shown to effectively alleviate salt and metal stress in plants by aiding in osmotic adjustment, ROS detoxification, and strengthening photosystem II structure.^33,34^

Excess B causes significant membrane damage. B is crucial for maintaining cell integrity, proliferation, signaling, and defense mechanisms, interacting with various essential enzymes and transcription factors.^35^ It is involved in synthesis and architecture.^36^ In tobacco and squash, 95–98% of B is located in the cell walls of leaves, cross-linked with glycosylinositol, phosphoceramides, and rhamnogalacturonan-II (RG-II), which regulates the cell wall’s tensile strength and absorbance.^37−40^ This remarkable ability of B to cross-link RG-II with borate esters makes B an indispensable element.^41^ It also contributes to better membrane integrity by participating in the protein and enzymatic activity of the cell membrane.^42^ The ideal B level increases plasma membrane hyperpolarization, but B deficiency alters membrane potential and reduces H^+^-ATPase activity.^43^ B also regulates ion (Ca^2+^, K^+^, PO_4_^3–^, H^+^, and Rb^+^) flow across membranes. Additionally, it plays an important role in nitrogen metabolism, carbohydrate metabolism and translocation, biosynthesis of phenols, nucleic acid metabolism, and regulation of hormone levels.^44^ Furthermore, B is essential for reproductive processes, especially pollen tube elongation, stimulation of reproductive tissues, and fruit and seed development.^45^ It also affects the uptake and availability of other elements from the soil. For example, a significant increase in the translocation and uptake of ions such as Cu, Fe, P, N, Zn, and K in seeds, leaves, and buds was observed after B treatment in cotton plants.^46^ Similarly, B deficiency reduces nitrate uptake by negatively affecting the activity of nitrate transporters in roots.^47^ Boron also plays vital functions in the metabolic activities of plants, and its importance in regulating the expansion of root and shoot meristems is related to the plant growth phase changes, which are critical processes in completing the life cycle of plants.^48^

Ancient wheat species, known for their genetic stability and resilience, offer a valuable yet often overlooked resource in modern agriculture. These hulled grains, such as Einkorn, Emmer, and Spelt, have been cultivated for millennia and possess unique nutritional profiles and resistance to various environmental stressors.^49−51^ Despite their potential benefits, their cultivation has declined due to factors like agricultural intensification, genetic erosion, and economic neglect.^52,53^ To address these challenges, research efforts should focus on understanding their agronomic characteristics, developing sustainable cultivation practices, and promoting their integration into diverse agricultural systems. Policy interventions are also crucial for supporting the conservation and utilization of these valuable genetic resources.

Numerous studies have been conducted to determine the nutritional content of hulled wheats,^54−56^ elucidate agronomic, morphological, and quality parameters,^51,57−61^ investigate anther culture,^62,63^ assess salt and drought tolerance,^64−67^ examine adaptation to climate change,^68−70^ explore pathogen resistance,^71^ reveal genetic diversity,^72,73^ and test the feasibility of producing less allergenic and more functional foods suitable for contemporary nutritional conditions.^61,74−76^ While extensive research has been conducted on the effects of B deficiency in plants,^71,77−80^ studies on the effects of excess boron and the molecular basis of these effects have been relatively limited. Recent studies have demonstrated that boron, a crucial component of the cell wall-plasma membrane-cytoskeleton continuum, also plays a role in intracellular signal transduction.^80^ However, there are still limited data on the mechanisms of boron exclusion from cells or its conversion into harmless forms under conditions of boron excess. Further studies of these mechanisms require the identification of boron-resistant varieties and a more detailed analysis of their genetic and molecular mechanisms. This study enables the observation of antioxidant defense mechanisms in ancestral hulled wheat varieties in response to boron excess and allows for the determination of whether these defense strategies facilitate survival under such conditions. The data obtained from this research will elucidate the mechanisms involved in combating boron excess.

Türkiye, as the origin of wheat, also possesses significant boron reserves. The widespread presence of boron processing facilities and boron-containing industrial products poses a growing threat to wheat cultivation. While wheat is sensitive to boron toxicity, ancient hulled wheat varieties, which are ancestors of modern wheat, may exhibit higher tolerance due to their genetic makeup. To assess this hypothesis, a laboratory-based study was conducted to evaluate the morphological, physiological, and antioxidant responses of four ancient hulled wheat species (*Triticum monococcum* L., *Triticum boeoticum* Boiss., *Triticum dicoccum* Schrank, and *Triticum speltoides* L.) to varying boron concentrations (0, 1, 5, 10, 15, and 20 mg L^–1^) and the mitigating effects of exogenously applied glycine-betaine (GB; 1 mM). Laboratory-based experiments provide a controlled environment to rapidly assess plant responses to stress factors, minimizing the time, labor, and financial resources required for field trials. By understanding the mechanisms of tolerance in ancient wheats to boron stress, we can develop strategies to enhance their resilience and promote their sustainable cultivation in boron-rich regions.

## 2 Materials and Methods

### 2.1 Plant Cultivation and Stress Management Practices

This study employed four distinct ancient hulled wheat varieties as the experimental materials. Boron concentrations of 5 mg L^–1^ and above are considered toxic for wheat. In this study, values were determined below this toxic B dose (1 mg L^–1^), at the threshold level (5 mg L^–1^), and above this dose (10, 15, and 20 mg L^–1^), based on B concentrations in Türkiye soils. Although B concentration in Türkiye soils generally ranges from 2 to 20 mg L^–1^, this ratio is at the level of 15–20 mg L^–1^, particularly in the soils of the Central Anatolia Region, where wheat cultivation is intensively practiced.^81^ The experimental protocol adhered to the methodology outlined by Temizgul^64^ and was conducted within an air-conditioned chamber. Plants were cultivated under stress-free conditions for a period of 10 days, followed by the imposition of boron stress for a subsequent 15 days. Control plants were nurtured exclusively with Hoagland solution^82^ for a total of 25 days, with boron being systematically removed from the solution. To prevent dehydration, the Hoagland solution was replenished frequently. The culture dishes containing the plants were refreshed daily, involving the removal of the old solution, excess water, and the subsequent addition of 25 mL of fresh Hoagland solution. Boron stress was applied at various concentrations of sodium borate (borax): 0, 1, 5, 10, 15, and 20 mg L^–1^ B (borax equivalent to 0 mg L^–1^, 9.53 mg L^–1^, 47.67 mg L^–1^, 95.34 mg L^–1^, 143.01 mg L^–1^, and 190.69 mg L^–1^, respectively). A separate set of treatments included the addition of 1 mM GB to each boron concentration: Control + 1 mM GB, 1 mg L^–1^ B + 1 mM GB, 5 mg L^–1^ B + 1 mM GB, 10 mg L^–1^ B + 1 mM GB, 15 mg L^–1^ B + 1 mM GB, and 20 mg L^–1^ B + 1 mM GB. Each experimental condition (12 sets) was replicated three times, resulting in a total of 36 samples. Upon completion of the 15-day stress period, plant roots and shoots were promptly harvested, flash-frozen in liquid nitrogen, and stored at −80 °C for subsequent analysis.

### 2.2 Plant Growth Measurements

Following a 15-day stress application period, fresh and dry weights of both roots and shoots were determined separately for all wheat samples by using an analytical balance. The length of the first emerging leaf, as well as the total plant, root, and shoot lengths, was measured with a ruler. To obtain dry weight measurements, samples were oven-dried at 65 °C for 48 h and subsequently weighed on a scale. The resulting values were recorded as the dry weight.

### 2.3 Determination of the Chlorophyll and Carotene Content

Chlorophyll and carotene content in the leaves were determined using the method described by Yilmaz et al.^83^ Briefly, 100 mg of fresh leaf samples was incubated in 10 mL of DMSO in a water bath at 65 °C until complete discoloration. The optical density of the resulting solution was measured at 647, 663, and 470 nm, using DMSO as a blank. Chlorophyll content was expressed as milligrams per gram fresh weight. The following formulas, adapted from Temizgul et al.,^84^ were used to calculate chlorophyll and carotene content.
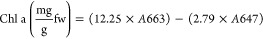

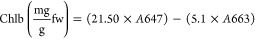

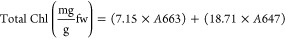

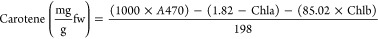


### 2.4 Enzyme Extraction and Protein Quantification

Crude enzyme extracts were prepared following the method described by Akbulut and Çakır^85^ for APX enzyme activity and the methods outlined by Yilmaz et al.^86^ for the activities of other enzymes. The crude extracts were stored at −20 °C until further use. Soluble protein concentrations were determined spectrophotometrically at 595 nm using the Bradford method^87^ with bovine serum albumin (BSA) fraction V as the standard. Soluble protein concentrations were expressed as μg mL^–1^.

### 2.5 Determination of Enzyme Activities

#### 2.5.1 Catalase (CAT) (EC 1.11.1.6)

Catalase activity of the samples was determined in a 20 mM sodium hydrogen phosphate (NaHPO_4_) buffer, pH 7.5, containing 15 mM H_2_O_2_ and 50 μL of crude enzyme extract, with modifications to the method of Duman et al.^88^ Measurements were conducted using a Shimadzu UV-1800 model cooled spectrophotometer (Shimadzu Corporation, Kyoto, Japan) in a 2 mL quartz cuvette. A buffer without the enzyme extract served as a blank. The reaction was initiated by adding H_2_O_2_ to the cuvette, and a decrease in absorbance was monitored for 3 min at 25 °C. For catalase, the molar absorption coefficient (ε) of H_2_O_2_ at 240 nm is 40 mM^–1^cm^–1^. Results were expressed as units per milligram of protein. Specific activity (SA) of the samples was calculated using the formula described by Temizgul et al.^84^
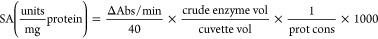


#### 2.5.2 Superoxide Dismutase (SOD) (EC 1.15.1.1)

To determine the SOD activity of the samples, the method described by Temizgul^64^ was employed. The reaction was conducted in 20 mM sodium phosphate buffer (pH 7.4) containing 0.1 mM ethylenediaminetetraacetic acid tetrasodium salt (Na_2_EDTA), 10 mM methionine, 0.1 mM nitro blue tetrazolium chloride (NBT), 0.005 mM riboflavin, and 50 μL of crude enzyme extract. The samples and standards were positioned 20 cm from a 500-lm fluorescent lamp for 15 min and then read against a blank at 560 nm using a spectrophotometer. Each experiment was replicated three times in duplicate. The SOD activity was calculated based on the percentage inhibition values of the samples. One unit of SOD activity is defined as the amount of enzyme required to inhibit the reaction by 50%. The percentage inhibition was calculated using the following formula:^84^
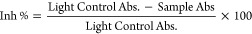


A logarithmic graph was plotted with the enzyme concentration against percentage inhibition. A new graph was constructed by taking the logarithms of the standard SOD enzyme concentrations and using the exact percentage inhibition values. The SOD concentrations were determined using the equation derived from this new graph. The results were expressed as units per milligram of protein.

#### 2.5.3 Ascorbate Peroxidase (APX) (EC 1.11.1.11)

APX activities of the samples were determined by adapting the method of Duman et al.^88^ A reaction mixture containing 50 mM potassium phosphate buffer (pH 7.0), 0.2 mM ascorbate, 10 mM H_2_O_2_, and 50 μL of crude enzyme extract was prepared. The reaction was initiated by adding H_2_O_2_ to a quartz cuvette maintained at 25 °C, and the change in activity was monitored spectrophotometrically at 290 nm for 3 min. Enzyme activity was calculated based on the amount of H_2_O_2_ consumed, utilizing the extinction coefficient (ε) of H_2_O_2_ (2.8 mM^–1^cm^–1^ at 290 nm).^84^ The results were expressed as units per milligram of protein.
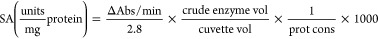


#### 2.5.4 Glutathione Reductase (GR) (EC 1.6.4.2)

GR activity was determined using the method described by Misra and Gupta.^89^ The reaction was carried out in a quartz cuvette at 25 °C for 5 min in a 100 mM potassium phosphate buffer (pH 7.5) containing 0.1 mM nicotinamide adenine dinucleotide phosphate (NADPH), 1 mM oxidized glutathione (GSSG), 0.1 mM Na_2_EDTA, and 50 μL of crude enzyme extract. The molar absorption coefficient (ε) of NADPH at 340 nm is 6.2 mM^–1^ cm^–1^. The specific activity of the samples was calculated using the formula provided by Temizgul et al.^84^ and expressed as units per milligram of protein.
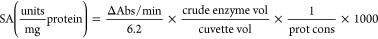


#### 2.5.5 Glutathione S-Transferase (GST) (EC 2.5.1.18)

GST activity was determined according to the method described by Yilmaz et al.^83^ The enzymatic reaction was carried out in a quartz cuvette at 25 °C and 340 nm for 5 min. The reaction mixture consisted of 100 mM potassium phosphate buffer (pH 7.5), 0.1 mM Na_2_EDTA, 0.1 mM NADPH, 1 mM glutathione (GSH), and 1 mM chloro-2,4-dinitrobenzene (CDNB). After adding 50 μL of crude enzyme extract to the reaction medium, a 5-min incubation period was allowed for nonspecific activity to cease. Subsequently, the absorbance change at 340 nm was monitored and recorded for an additional 5 min. The molar absorption coefficient (ε) of NADPH at 340 nm is 6.2 mM^–1^ cm^–1^. The specific activity of the samples was calculated using the formula provided by Temizgul et al.^84^ and expressed as units per milligram of protein.
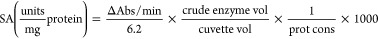


### 2.6 Determination of the Proline Accumulation

Proline content was determined according to the method described by Temizgul et al.^84^ This involved reacting samples with acid ninhydrin in sulfosalicylic acid, extracting the resulting color complex with toluene, and measuring the absorbance at 520 nm against a toluene blank using a spectrophotometer. In addition to the samples, a standard curve was generated using 10 proline standards ranging from 0.01 μM to 1.5 mM. The equation derived from this standard curve was employed to calculate the proline content of the samples, which was expressed as nmol g^–1^ fresh weight.

### 2.7 Lipid Peroxidation Analysis (MDA) (LPO)

To assess the impact of boron applications on lipid peroxidation, the thiobarbituric acid reactive substances (TBARS) content in the tissues was evaluated using a 20% trichloroacetic acid (TCA) solution containing 0.5% 2-thiobarbituric acid (TBA), following the method described by Madhava and Sresty.^90^ The results were expressed as TBARS (nmol g^–1^ fresh weight). The TBARS analysis quantifies malondialdehyde (MDA), a product of lipid peroxidation, both directly from the samples and indirectly from lipid hydroperoxides that are hydrolyzed during the reaction.^91^

### 2.8 Statistical Evaluation of the Results

A comprehensive statistical analysis was conducted to evaluate the impact of B stress on different wheat varieties and plant parts. Two-way and three-way analyses of variance (ANOVA) were performed using SPSS 28.0 and GraphPad Prism version 10.0.0, respectively. The two-way ANOVA, conducted in SPSS 28.0, assessed the interactions between B doses and wheat varieties, as well as B doses and plant parts. The three-way ANOVA, performed using GraphPad Prism, examined the combined effects of B doses, plant parts (roots and shoots), and wheat varieties (*T. monococcum*, *T. dicoccum*, *T. speltoides*, and *T. boeoticum*). Multiple comparison tests, including the least significant difference (LSD) test and Tukey test, were employed to further determine significant differences among treatment groups. Additionally, “Tests of Between-Subjects Effects”, “Levene’s Test of Equality of Error Variances”, and “MANOVA-Multivariate Tests” were conducted to elucidate the effects of B applications. All statistical analyses were performed at a significance level of *p* ≤ 0.05. Each experiment was replicated three times, and data were presented as the mean ± standard deviation (SD). Different lowercase letters (a, b, c, d, and e) within tables and figures indicate statistically significant differences between means.

## 3 Results

### 3.1 Effect of Boron Application on Morphological Indicators

**Figure 1:**
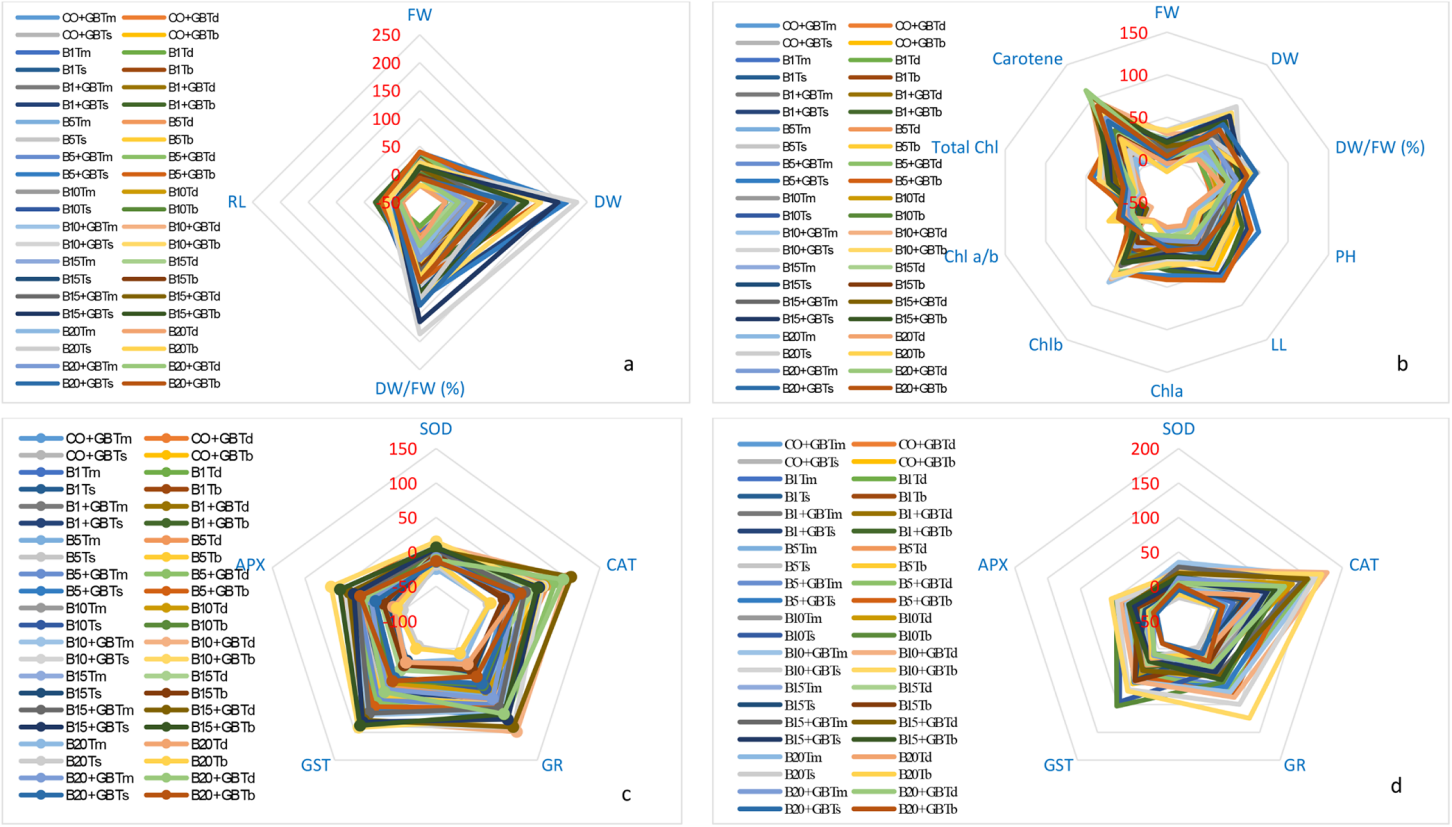
Percentage changes in morphological and antioxidant responses observed in hulled wheat depending on borax and GB applications compared to the control. (a) Root-morphological, (b) shoot-morphological, (c) root-antioxidant, (d) shoot-antioxidant. CO: Control; GB: Glycine-betaine; B: Boron; B1:1 mg L^–1^ B; B5:5 mg L^–1^ B; B10:10 mg L^–1^ B; B15:15 mg L^–1^ B; B20:20 mg L^–1^ B treatments; GB: Glycine betaine (1 mM); Tm: *T. monococcum*; Td: *T. dicoccum*; Ts: *T. speltoides*; Tb: *T. boeoticum*.

**Figure 2:**
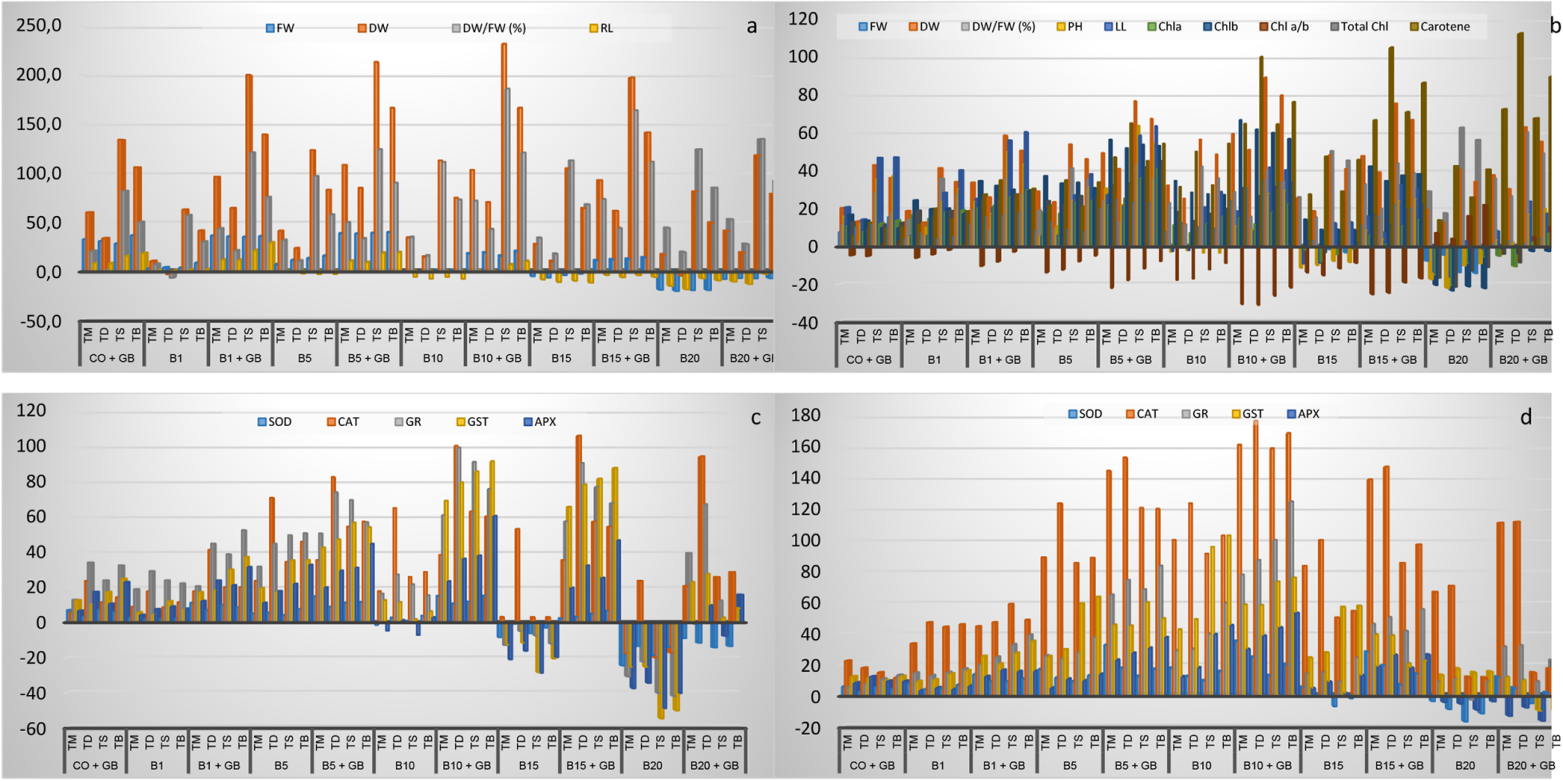
Percentage changes in morphological and antioxidant responses observed in hulled wheat depending on borax and GB applications compared to the control (column chart representation). (a) Root-morphological; (b) shoot-morphological; (c) root-antioxidant; (d) shoot-antioxidant. CO: Control; B1:1 mg L^–1^ B; B5:5 mg L^–1^ B; B10:10 mg L^–1^ B; B15:15 mg L^–1^ B; B20:20 mg L^–1^ B; GB: Glycine betaine (1 mM); Tm: *T. monococcum*; Td: *T. dicoccum*; Ts: *T. speltoides*; Tb: *T. boeoticum*.

Tables 1 and 2 present the morphological changes observed in hulled wheat as a function of B application. B application led to a consistent increase in wet root weight up to a concentration of 10 mg L^–1^. The increase in fresh root weight varied significantly among wheat varieties (*p* ≤ 0.05). While *Triticum boeoticum* exhibited a 15.9% increase compared to the control, *Triticum monococcum* showed only a 7.1% increase (Figure 2a). Beyond 10 mg L^–1^ B, a significant weight reduction was observed (Tables 1 and S1). In contrast to the fresh weight, the dry root weight continued to increase with increasing B doses. At 20 mg L^–1^ B, *T. speltoides* exhibited an 81.6% increase in root dry weight compared to the control (Figure 2a). When B applications were supplemented with exogenous GB, root fresh weight increased by approximately 15% at 15 mg L^–1^ B and decreased by approximately 7% at 20 mg L^–1^ B (Table 1, Figure 2a). Root dry weight doubled in B applications supplemented with GB compared to the control. The DW/FW ratio increased by 27.9% in *T. dicoccum*and 135% in *T. boeoticum* in GB-supported B applications (Figures 1a and 2a). B application shortened the wheat root length by 1–18%.

**Table 1:** Effects of Borax and GB Applications on the Root Development of Hulled Wheats^i^^,^^ii^^,^iii

Applications	Wheats	FW (g)	DW (g)	DW/FW (%)	RL (cm)
Control	Tm	4.24 ± 0.15^d^	0.58 ± 0.03^h^	13.68 ± 0.54^g^	8.08 ± 0.36^bc^
	Td	4.23 ± 0.15^d^	**0.68 ± 0.03**^**g**^	**16.08 ± 0.64**^**ef**^	**8.26 ± 0.37**^**bc**^
	Ts	4.22 ± 0.15^d^	0.38 ± 0.02^j^	9.01 ± 0.36^i^	7.44 ± 0.33^cd^
	Tb	**4.28 ± 0.15**^**d**^	0.48 ± 0.02^i^	11.22 ± 0.44^h^	7.65 ± 0.34^c^
Control +1 mM GB	Tm	5.63 ± 0.19^a^	0.93 ± 0.05^d^	16.52 ± 0.66^ef^	8.76 ± 0.39^b^
	Td	5.54 ± 0.19^a^	0.91 ± 0.05^d^	16.43 ± 0.65^ef^	8.94 ± 0.40^b^
	Ts	5.42 ± 0.18^b^	0.89 ± 0.05^e^	16.42 ± 0.65^ef^	8.63 ± 0.38^b^
	Tb	**5.86 ± 0.20**^**a**^	**0.99 ± 0.05**^**d**^	**16.89 ± 0.67**^**ef**^	**9.07 ± 0.40**^**ab**^
5 mg L^-1^ Borax	Tm	4.54 ± 0.15^c^	0.82 ± 0.04^e^	**18.06 ± 0.72**^**de**^	8.02 ± 0.36^bc^
	Td	4.71 ± 0.16^c^	0.84 ± 0.04^e^	17.83 ± 0.71^e^	**8.08 ± 0.36**^**bc**^
	Ts	4.78 ± 0.16^c^	0.85 ± 0.04^e^	17.78 ± 0.71^e^	7.24 ± 0.32^cd^
	Tb	**4.96 ± 0.17**^**c**^	**0.88 ± 0.04**^**e**^	17.74 ± 0.70^e^	7.45 ± 0.33^cd^
5 mg L^-1^ Borax +1 mM GB	Tm	5.89 ± 0.20^a^	1.21 ± 0.06^a^	20.54 ± 0.82^cd^	8.94 ± 0.40^b^
	Td	5.86 ± 0.20^a^	1.26 ± 0.06^a^	**21.50 ± 0.86**^**c**^	9.03 ± 0.40^ab^
	Ts	5.88 ± 0.20^a^	1.19 ± 0.06^b^	20.24 ± 0.80^cd^	8.85 ± 0.39^b^
	Tb	**5.99 ± 0.20**^**a**^	**1.28 ± 0.06**^**a**^	21.37 ± 0.85^c^	**9.14 ± 0.41**^**ab**^

i FW: Fresh weight; DW: Dry weight; RL: Root length; GB: Glycine-Betaine; Tm: *T. monococcum*; Td: *T. dicoccum*; Ts: *T. speltoides*; Tb: *T. boeoticum*.

ii The table containing all data are given in Table S1.

iii Differences in the letters indicates statistical significance at 5% level in the columns.

**Table 2:** Effects of Borax and GB Applications on the Stem Development of Hulled Wheats^i^^,^^ii^

Applications	Wheats	FW (g)	DW (g)	DW/FW (%)	PH (cm)	LL (cm)	Chl_a (mg g^–1^ fw)	Chl_b (mg g^–^^1^ fw)	Chl a/b	Total Chl (mg g^–^^1^ fw)	Carotene (mg g^–^^1^ fw)
Control	Tm	28.28 ± 1.27^gh^	3.79 ± 0.17^de^	13.40 ± 0.60^cd^	31.59 ± 1.42^d^	18.67 ± 0.84^f^	1.81 ± 0.08^cd^	0.78 ± 0.03^d^	2.32 ± 0.10^b^	2.59 ± 0.11^fg^	0.51 ± 0.02^d^
	Td	27.22 ± 1.22^h^	3.98 ± 0.17^de^	14.62 ± 0.65^c^	35.11 ± 1.57^c^	20.54 ± 0.92^e^	1.90 ± 0.08^c^	0.81 ± 0.03^cd^	**2.35 ± 0.10**^**b**^	2.71 ± 0.12^ef^	0.40 ± 0.01^e^
	Ts	30.98 ± 1.39^fg^	3.21 ± 0.14^ef^	10.36 ± 0.46^e^	25.74 ± 1.15^ef^	16.80 ± 0.75^g^	1.71 ± 0.07^d^	0.80 ± 0.03^cd^	2.14 ± 0.09^c^	2.51 ± 0.11^fg^	0.62 ± 0.02^cd^
	Tb	31.36 ± 1.41^f^	3.40 ± 0.15^e^	10.84 ± 0.48^e^	28.08 ± 1.26^de^	17.73 ± 0.79^fg^	1.67 ± 0.07^de^	0.81 ± 0.03^cd^	2.06 ± 0.09^cd^	2.48 ± 0.11^g^	0.59 ± 0.02^d^
Control +1 mM GB	Tm	30.44 ± 1.36^fg^	4.56 ± 0.20^cd^	14.98 ± 0.67^bc^	36.65 ± 1.64^bc^	22.54 ± 1.01^d^	2.01 ± 0.09^bc^	0.91 ± 0.04^c^	2.21 ± 0.09^bc^	2.92 ± 0.13^de^	0.54 ± 0.02^d^
	Td	30.02 ± 1.35^fg^	4.51 ± 0.20^cd^	15.02 ± 0.67^bc^	**39.32 ± 1.76**^**b**^	23.46 ± 1.05^cd^	2.05 ± 0.09^bc^	0.92 ± 0.04^c^	2.23 ± 0.10^bc^	2.97 ± 0.13^de^	0.45 ± 0.02^e^
	Ts	34.56 ± 1.55^de^	4.59 ± 0.20^cd^	13.28 ± 0.59^cd^	34.87 ± 1.56^c^	24.67 ± 1.11^c^	1.92 ± 0.08^c^	0.88 ± 0.03^cd^	2.18 ± 0.09^c^	2.80 ± 0.12^e^	0.68 ± 0.03^cd^
	Tb	36.22 ± 1.62^cd^	4.63 ± 0.20^cd^	12.78 ± 0.57^d^	38.54 ± 1.73^b^	**26.08 ± 1.17**^**b**^	1.90 ± 0.08^c^	0.89 ± 0.04^cd^	2.13 ± 0.09^c^	2.79 ± 0.12^ef^	0.65 ± 0.02^cd^
10 mg L^-1^ Borax	Tm	30.44 ± 1.36^fg^	5.01 ± 0.22^c^	16.45 ± 0.74^b^	30.72 ± 1.38^d^	18.21 ± 0.81^f^	2.01 ± 0.09^bc^	1.05 ± 0.04^bc^	1.91 ± 0.08^d^	3.06 ± 0.13^d^	0.67 ± 0.03^cd^
	Td	30.56 ± 1.37^fg^	4.98 ± 0.22^c^	16.29 ± 0.73^b^	34.44 ± 1.54^c^	20.14 ± 0.90^e^	2.03 ± 0.09^bc^	1.04 ± 0.04^bc^	1.95 ± 0.08^d^	3.07 ± 0.13^d^	0.60 ± 0.02^cd^
	Ts	34.08 ± 1.53^de^	5.02 ± 0.22^c^	14.73 ± 0.66^c^	24.91 ± 1.12^ef^	20.26 ± 0.91^e^	1.92 ± 0.08^c^	1.02 ± 0.04^bc^	1.88 ± 0.08^de^	2.94 ± 0.13^de^	0.82 ± 0.03^bc^
	Tb	34.25 ± 1.54^de^	5.05 ± 0.22^bc^	14.74 ± 0.66^c^	27.16 ± 1.22^e^	22.84 ± 1.02^d^	1.94 ± 0.08^c^	1.03 ± 0.04^bc^	1.88 ± 0.08^de^	2.97 ± 0.13^de^	0.91 ± 0.04^b^
10 mg L^-1^ Borax +1 mM GB	Tm	35.02 ± 1.57^d^	6.04 ± 0.27^a^	**17.24 ± 0.77**^**ab**^	35.08 ± 1.57^c^	22.12 ± 0.99^d^	2.09 ± 0.09^bc^	1.30 ± 0.05^a^	1.61 ± 0.07^ef^	3.39 ± 0.15^bc^	0.84 ± 0.03^bc^
	Td	35.54 ± 1.59^d^	6.01 ± 0.27^a^	16.91 ± 0.76^b^	38.24 ± 1.72^b^	22.28 ± 1.00^d^	**2.12 ± 0.09**^**b**^	**1.31 ± 0.05**^**a**^	1.62 ± 0.07^ef^	**3.43 ± 0.15**^**b**^	0.80 ± 0.03^bc^
	Ts	41.78 ± 1.88^a^	6.07 ± 0.27^a^	14.52 ± 0.65^c^	32.96 ± 1.48^cd^	23.81 ± 1.07^cd^	2.02 ± 0.09^bc^	1.28 ± 0.05^ab^	1.58 ± 0.07^f^	3.30 ± 0.14^bc^	1.02 ± 0.04^ab^
	Tb	**41.91 ± 1.88**^**a**^	**6.11 ± 0.27**^**a**^	14.57 ± 0.65^c^	36.41 ± 1.63^bc^	24.87 ± 1.11^c^	2.05 ± 0.09^bc^	1.27 ± 0.05^ab^	1.61 ± 0.07^ef^	3.32 ± 0.14^bc^	**1.04 ± 0.04**^**ab**^

i FW: Fresh weight; DW: Dry weight; PH: Plant height; LL: Leaf length; GB: Glycine-Betaine; Tm: *T. monococcum*; Td: *T. dicoccum*; Ts: *T. speltoides*; Tb: *T. boeoticum*.

ii The table containing all data is given in Table S2. Differences in the letters indicates statistical significance at 5% level in the columns.

B application led to increases in fresh and dry weight, plant height, and leaf length of wheat shoots up to a dose of 10 mg L^–1^. However, B applications of 15 mg L^–1^ and above caused a decrease in all measured morphological parameters in wheat shoots (Tables 2, S2, and Figure 2b). Chlorophyll a and b content increased up to 15 mg L^–1^ B, with a more pronounced increase in chlorophyll b (chl_a 15%, chl_b 67%). This increase in chlorophyll b content negatively affected the chlorophyll a/b ratio. Carotene content in wheat increased continuously (5–112%) with increasing B application (Table 2, Figures 1b and 2b). Based on the morphological data obtained from roots and shoots at high B doses (20 mg L^–1^), *T. speltoides* and *T. boeoticum* were more tolerant than other hulled wheat varieties (Figure 2a,b).

### 3.2 Effect of B Applications on Total Soluble Protein Content

**Figure 3:**
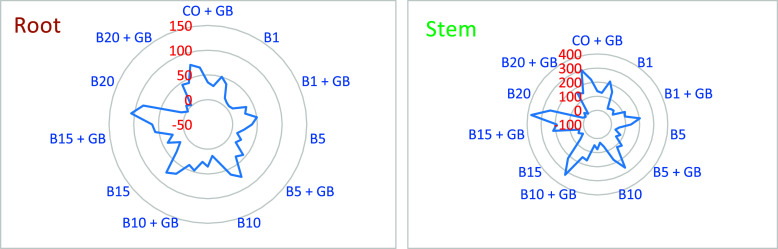
Percentage change in total soluble protein accumulation compared to the control. CO: Control; GB: Glycine-betaine; B: Boron; B1:1 mg L^–1^ B; B5:5 mg L^–1^ B; B10:10 mg L^–1^ B; B15:15 mg L^–1^ B; B20:20 mg L^–1^ B treatments.

While B applications generally caused a 10–100% increase in soluble protein content in hulled wheat roots, this increase reached 300% in wheat shoots (Tables 3, 4 and S3, S4). Exogenous GB application generally increased protein accumulation in wheat roots and shoots by up to 3-fold (Figure 3a,b).

**Table 3:** Antioxidant Responses Caused by Borax and GB Applications in the Root of Hulled Wheats^i^^,^^ii^

Applications	Wheats	TPC (μg mL^–1^ protein)	SOD (U mL^–1^ protein)	CAT (U mL^–1^ protein)	GR (U mL^–1^ protein)	GST (U mL^1^ protein)	APX (U mL^1^ protein)	PRO (nmol g^–1^ fw)	MDA (nmol g^–1^ fw)
Control	Tm	500.05 ± 22.50^gh^	1.118 ± 0.05^ef^	0.034 ± 0.001^e^	0.117 ± 0.005^d^	0.087 ± 0.004^e^	0.538 ± 0.024^de^	23.55 ± 1.06^e^	7.35 ± 0.33^e^
	Td	548.47 ± 24.68^g^	1.172 ± 0.05^de^	0.017 ± 0.001^h^	0.103 ± 0.005^d^	0.087 ± 0.004^e^	0.478 ± 0.022^e^	26.50 ± 1.19^e^	5.99 ± 0.27^e^
	Ts	415.32 ± 18.68^i^	1.254 ± 0.05^cd^	0.035 ± 0.001^de^	0.176 ± 0.008^c^	0.173 ± 0.008^cd^	0.637 ± 0.029^cd^	36.75 ± 1.65^de^	8.24 ± 0.37^e^
	Tb	449.90 ± 20.24^hi^	1.207 ± 0.05^d^	0.035 ± 0.001^de^	0.176 ± 0.008^c^	0.161 ± 0.007^cd^	0.558 ± 0.025^d^	31.63 ± 1.42^e^	7.49 ± 0.34^e^
Control +1 mM GB	Tm	675.28 ± 30.38^e^	1.196 ± 0.05^de^	0.036 ± 0.002^de^	0.132 ± 0.006^cd^	0.098 ± 0.004^de^	0.574 ± 0.026^d^	32.70 ± 1.47^e^	6.64 ± 0.30^e^
	Td	705.26 ± 31.73^de^	1.208 ± 0.05^d^	0.021 ± 0.001^g^	0.138 ± 0.006^cd^	0.096 ± 0.004^de^	0.561 ± 0.025^d^	32.88 ± 1.48^e^	5.90 ± 0.27^e^
	Ts	623.98 ± 28.07^ef^	1.304 ± 0.06^b^	0.039 ± 0.002^d^	0.218 ± 0.010^bc^	0.203 ± 0.009^c^	0.705 ± 0.032^bc^	41.13 ± 1.85^d^	6.42 ± 0.29^e^
	Tb	626.28 ± 28.18^ef^	1.278 ± 0.05^c^	0.040 ± 0.002^d^	0.233 ± 0.010^bc^	0.201 ± 0.009^c^	0.686 ± 0.031^c^	38.41 ± 1.72^de^	6.48 ± 0.29^e^
15 mg L^-1^ Borax	Tm	664.90 ± 29.92^e^	1.024 ± 0.04^g^	0.035 ± 0.002^de^	0.102 ± 0.005^d^	0.076 ± 0.003^e^	0.425 ± 0.019^ef^	61.03 ± 2.74^cd^	35.63 ± 1.60^bc^
	Td	638.96 ± 28.75^ef^	1.163 ± 0.05^e^	0.026 ± 0.001^f^	0.098 ± 0.004^d^	0.077 ± 0.003^e^	0.401 ± 0.018^ef^	76.40 ± 3.43^cd^	36.09 ± 1.62^bc^
	Ts	578.45 ± 26.03^fg^	1.177 ± 0.05^de^	0.036 ± 0.002^de^	0.163 ± 0.007^c^	0.124 ± 0.006^d^	0.454 ± 0.020^e^	84.35 ± 3.79^c^	**45.77 ± 2.06**^**b**^
	Tb	558.27 ± 25.12^g^	1.171 ± 0.05^de^	0.036 ± 0.002^de^	0.155 ± 0.007^cd^	0.128 ± 0.006^d^	0.448 ± 0.020^ef^	78.56 ± 3.53^c^	45.13 ± 2.03^b^
15 mg L^-1^ Borax +1 mM GB	Tm	787.68 ± 35.44^c^	1.143 ± 0.05^e^	0.046 ± 0.002^c^	0.184 ± 0.008^c^	0.144 ± 0.006^d^	0.644 ± 0.029^cd^	112.54 ± 5.06^bc^	13.36 ± 0.60^de^
	Td	**891.43 ± 40.11**^**a**^	1.208 ± 0.05^d^	0.035 ± 0.001^de^	0.196 ± 0.009^c^	0.155 ± 0.007^cd^	0.633 ± 0.028^cd^	128.06 ± 5.76^b^	13.68 ± 0.62^de^
	Ts	858.01 ± 38.61^ab^	**1.314 ± 0.05**^**bc**^	**0.055 ± 0.003**^**ab**^	**0.311 ± 0.014**^**ab**^	**0.314 ± 0.014**^**a**^	0.799 ± 0.036^ab^	**163.84 ± 7.37**^**a**^	16.28 ± 0.73^d^
	Tb	837.25 ± 37.67^b^	1.285 ± 0.05^c^	0.054 ± 0.002^ab^	0.295 ± 0.013^ab^	0.302 ± 0.014^a^	**0.818 ± 0.037**^**ab**^	160.48 ± 7.22^a^	15.84 ± 0.71^d^

i TPC: Total soluble protein content; PRO: Proline; GB: Glycine-Betaine; Tm: *T. monococcum*; Td: *T. dicoccum*; Ts: T. speltoides; Tb: *T. boeoticum*.

ii The table containing all data is given in Table S3. Differences in the letters indicates statistical significance at 5% level in the columns.

**Table 4:** Antioxidant Responses Caused by Borax and GB Applications in the Shoot of Hulled Wheats^i^^,^^ii^

Applications	Wheats	TPC (μg mL^–1^ protein)	SOD (U mL^–1^ protein)	CAT (U mL^1^ protein)	GR (U mL^–1^ protein)	GST (U mL^–1^ protein)	APX (U mL^–1^ protein)	PRO (nmol g^–1^ fw)	MDA (nmol g^–1^ fw)
Control	Tm	271.22 ± 12.20^fg^	0.962 ± 0.043^g^	0.018 ± 0.001^e^	0.117 ± 0.005^e^	0.099 ± 0.004^e^	0.577 ± 0.026^e^	21.45 ± 0.97^g^	6.52 ± 0.29^f^
	Td	298.31 ± 13.42^f^	1.014 ± 0.046^f^	0.017 ± 0.001^e^	0.117 ± 0.005^e^	0.098 ± 0.004^e^	0.537 ± 0.024^ef^	24.78 ± 1.12^g^	5.62 ± 0.25^f^
	Ts	185.33 ± 8.34^g^	1.144 ± 0.051^cd^	0.034 ± 0.002^d^	0.161 ± 0.007^de^	0.135 ± 0.006^de^	0.637 ± 0.029^d^	32.05 ± 1.44^g^	7.12 ± 0.32^e^
	Tb	233.17 ± 10.49^g^	1.062 ± 0.048^e^	0.035 ± 0.002^d^	0.146 ± 0.007^de^	0.137 ± 0.006^de^	0.617 ± 0.028^de^	30.34 ± 1.37^g^	6.74 ± 0.30^f^
Control +1 mM GB	Tm	641.27 ± 28.86^ab^	1.018 ± 0.046^f^	0.022 ± 0.001^e^	0.124 ± 0.006^e^	0.111 ± 0.005^e^	0.624 ± 0.028^d^	24.36 ± 1.10^g^	5.44 ± 0.24^f^
	Td	674.70 ± 30.36^ab^	1.039 ± 0.047^e^	0.020 ± 0.001^e^	0.126 ± 0.006^e^	0.109 ± 0.005^e^	0.602 ± 0.027^de^	30.47 ± 1.37^g^	5.02 ± 0.23^f^
	Ts	588.82 ± 26.50^b^	1.205 ± 0.054^bc^	0.039 ± 0.002^d^	0.178 ± 0.008^d^	0.148 ± 0.007^d^	0.696 ± 0.031^c^	58.36 ± 2.63^f^	6.25 ± 0.28^f^
	Tb	589.39 ± 26.52^b^	1.107 ± 0.050^d^	0.039 ± 0.002^d^	0.165 ± 0.007^d^	0.154 ± 0.007^d^	0.674 ± 0.030^cd^	57.88 ± 2.60^f^	5.86 ± 0.26^f^
15 mg L^-1^ Borax	Tm	435.49 ± 19.60^d^	1.013 ± 0.046^f^	0.033 ± 0.001^d^	0.134 ± 0.006^e^	0.123 ± 0.006^de^	0.603 ± 0.027^de^	86.22 ± 3.88^de^	27.32 ± 1.23^bc^
	Td	364.60 ± 16.41^e^	1.016 ± 0.046^f^	0.034 ± 0.002^d^	0.135 ± 0.006^e^	0.125 ± 0.006^de^	0.584 ± 0.026^de^	91.38 ± 4.11^de^	31.63 ± 1.42^b^
	Ts	275.83 ± 12.41^f^	1.066 ± 0.048^de^	0.051 ± 0.002^c^	0.176 ± 0.008^d^	0.212 ± 0.010^b^	0.645 ± 0.029^cd^	124.68 ± 5.61^c^	**32.55 ± 1.46**^**b**^
	Tb	309.26 ± 13.92^f^	1.044 ± 0.047^e^	0.054 ± 0.002^c^	0.182 ± 0.008^d^	**0.216 ± 0.010**^**b**^	0.696 ± 0.031^c^	146.57 ± 6.60^b^	30.87 ± 1.39^b^
15 mg L^-1^ Borax +1 mM GB	Tm	862.25 ± 38.80^a^	**1.232 ± 0.055**^**b**^	0.043 ± 0.002^cd^	0.171 ± 0.008^d^	0.138 ± 0.006^d^	0.684 ± 0.031^cd^	102.98 ± 4.63^d^	12.22 ± 0.55^c^
	Td	864.92 ± 38.92^a^	1.210 ± 0.054^bc^	0.042 ± 0.002^cd^	0.176 ± 0.008^d^	0.136 ± 0.006^de^	0.678 ± 0.031^cd^	109.36 ± 4.92^cd^	12.38 ± 0.56^c^
	Ts	**879.33 ± 39.57**^**a**^	1.225 ± 0.055^b^	0.063 ± 0.003^bc^	**0.228 ± 0.010**^**c**^	0.163 ± 0.007^cd^	0.751 ± 0.034^bc^	146.54 ± 6.59^b^	11.65 ± 0.52^c^
	Tb	813.38 ± 36.60^a^	1.211 ± 0.054^bc^	**0.069 ± 0.003**^**b**^	0.227 ± 0.010^c^	0.168 ± 0.008^cd^	**0.779 ± 0.035**^**b**^	**163.08 ± 7.34**^**ab**^	11.24 ± 0.51^c^

i TPC: Total soluble protein content; PRO: Proline; GB: Glycine-Betaine; Tm: *T. monococcum*; Td: *T. dicoccum*; Ts: *T. speltoides*; Tb: *T. boeoticum*.

ii The table containing all data is given in Table S4. Differences in the letters indicates statistical significance at 5% level in the columns.

### 3.3 Effects of B Application on Antioxidant Enzyme Activity

Tables 3 and 4 present the antioxidant enzyme activities of hulled wheat in response to B treatments. The highest antioxidant enzyme activities were observed at 5 mg L^–1^ B. However, exogenous GB application significantly increased antioxidant enzyme activities at 15 and 20 mg L^–1^ B (Tables 3, 4 and S3, S4; Figures 1c,d, and 2c,d). The increase in antioxidant enzyme activities was generally higher in wheat stems (shoots) than in roots (Figures 1c,d, and 2c,d).

Significant decreases in enzyme activities were observed in roots at 20 mg L^–1^ B (SOD 10–25%, CAT 10–20%, GR 20–40%, GST 25–50%, and APX 25–50%). However, when 20 mg L^–1^ B was supplemented with exogenous GB, a significant increase in antioxidant enzyme activity was observed in wheat roots (SOD 8–14%, CAT 20–90%, GR 10–40%, GST 10–25%, and APX 0–15%) (Figure 2c). At 20 mg L^–1^ B, SOD and APX activities decreased in wheat shoots (3–16% and 3–9%, respectively), while CAT, GR, and GST activities increased (11–70%, 0–11%, and 13–17%, respectively; Table 4). Interestingly, unlike roots, exogenous GB application did not significantly increase enzyme activity in shoots and even caused a decrease in GST and APX activities (except for CAT) (Tables 4 and S4; Figure 2d).

### 3.4 Effect on Proline Accumulation

**Figure 4:**
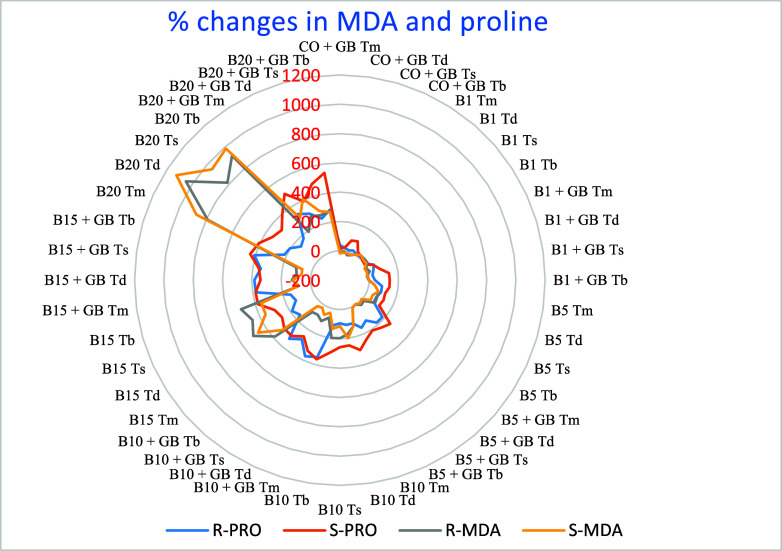
Percentage changes in MDA and proline contents compared with the control. R-PRO: Root proline; S-PRO: Shoot proline; R-MDA: Root MDA; S-MDA: Shoot MDA; CO: Control; GB: Glycine-betaine (1 mM); B: Boron; B1:1 mg L^–1^ B; B5:5 mg L^–1^ B; B10:10 mg L^–1^ B; B15:15 mg L^–1^ B; B20:20 mg L^–1^ B treatments; Tm: *T. monococcum*; Td: *T. dicoccum*; Ts: *T. speltoides*; Tb: *T. boeoticum*.

B applications resulted in proline accumulation ranging from 23 to 163 nmol g^–1^ FW in roots and 20 to 194 nmol g^–1^ FW in the shoots (Tables 3 and 4). The percentage increase in proline accumulation in shoots compared to the control was higher than that in roots (300–400% in roots and 400–550% in shoots) (Figure 4).

### 3.5 Effect on Lipid Peroxidation (LPO) (MDA)

B applications resulted in MDA levels ranging from 5.99 to 76.62 nmol gr^–1^ FW in roots and 5.62 to 73.28 nmol gr^–1^ FW in shoots (Tables 3, 4 and S3, S4). At 20 mg L^–1^ B, MDA increased by 800–1050% in roots and 875–1124% in shoots compared with the control. Exogenous GB application reduced MDA levels to 200–350% in roots and 268–409% in shoots at 20 mg L^–1^ B (Figures 4).

### 3.6 ANOVA, MANOVA, Multiple Comparisons, and Levene Test Results

No significant differences were observed in soluble protein concentration (*p* ≤ 0.106), SOD (*p* ≤ 0.175), and GST (*p* ≤ 0.205) enzyme activities between roots and shoots. The antioxidant responses of wheat to different B doses did not show significant differences in CAT (*p* ≤ 0.179) and GST (*p* ≤ 0.268) enzyme activities. Additionally, there were no significant differences in CAT (*p* ≤ 0.334) and GST (*p* ≤ 0.501) activities among plant parts under different B doses. When all variables were considered together (Wheat × Parts × Doses), significant differences were observed among other factors, except for CAT (*p* ≤ 0.449), GR (*p* ≤ 0.139), and GST (*p* ≤ 0.311) enzyme activities (Table S5).

Levene’s test, a statistical test used to assess the homogeneity of variances, evaluates the null hypothesis that the error variance of the dependent variable is equal across groups. The result of Levene’s test allows us to determine whether the difference between groups is statistically significant. If the Levene’s test result is significant, it indicates that there is variance inequality between groups, and in this case, we may need to use different analysis methods. In our study, the *p*-values of all tested variables were >0.05, indicating that there was no variance inequality between groups and that there was no need for another test tool. Levene’s test showed significant differences in the activities of all antioxidant enzymes (*p* ≤ 0.001) (Table S6).

Multiple comparison analyses revealed no significant difference between *T. speltoides* and *T. boeoticum* in terms of soluble protein concentrations; between *T. monococcum* and *T. dicoccum* and between *T. speltoides* and *T. boeoticum*in terms of CAT activity; between *T. monococcum* and *T. dicoccum* in terms of GR activity; and between *T. dicoccum* and *T. boeoticum* in terms of GST enzyme activities (*p* ≤ 0.196, *p* ≤ 0.651, *p* ≤ 0.412, *p* ≤ 0.279, and *p* ≤ 0.104, respectively) (Table S7).

The MANOVA multivariate test generates four test statistics (Pillai’s Trace, Wilks’ Lambda, Hotelling’s Trace, and Roy’s Largest Root), and if *p* ≥ 0.05 in the tested factors, these test statistics are examined. In this study, although the *F*-values for each test statistic varied, the null hypothesis of MANOVA was rejected because *p* ≤ 0.05 (*p* ≤ 0.001), and it was concluded that the wheat variety, the plant parts, and the B doses were important in the enzymatic/nonenzymatic antioxidant response (Table S8). This test revealed significant differences in enzyme activities across all applications (Wheat × Parts × Doses) (Pillai’s Trace, V: 2.566, F: 2.65, *p* ≤ 0.001; Wilks’ Lambda, V: 0.030, F: 3.040, *p* ≤ 0.001; Hotelling’s Trace, V: 5.237, F: 3.567, *p* ≤ 0.001; and Roy’s Largest Root, V: 2.272, F: 12.746, *p* ≤ 0.001) (Table S8).″

## 4 Discussion

### 4.1 Effects of Boron on Plant Morphology

Stimulating shoot development can reduce the level of B uptake. Akçay and Erkan^92^ suggested that this decrease in shoot B concentration is a dilution effect resulting from increased plant tissue. Koohkan and Maftoun^93,94^ observed similar results with nitrogen application in rice and canola, attributing the decreased seed B concentration to growth-induced dilution. In our study, exogenous GB application increased shoot biomass, mitigating B toxicity symptoms. Anthocyanins can chelate B ions, potentially reducing their toxicity,^95,96^ although direct evidence of this mechanism is lacking. Landi et al.^96^ suggested that foliar anthocyanin application mitigated B toxicity in sweet basil. de Souza et al.^97^ proposed that B immobilization in roots prevents translocation to shoots, thereby reducing B accumulation in sensitive shoot tissues. Aftab et al.^98^ linked high B tolerance to increased root B accumulation and decreased shoot B transport. *T. speltoides* and *T. boeoticum* showed 81% and 50% greater root dry matter, respectively, than the control at 20 mg L^–1^ B. Exogenous GB further increased root dry matter, reaching 118.4% and 79.2% above the control, respectively. This increase suggests a plant strategy to protect shoots from B stress. *T. speltoides* also showed a 124% increase in the root dry weight/fresh weight (dw/fw) ratio at 20 mg L^–1^ B, with a 62% increase in the shoot ratio (Table 2; Figure 2a,b). B is primarily transported via transpiration and accumulates in above-ground tissues, causing damage.^45^ Thus, B tolerance often involves restricting B translocation from root to shoot.^99^

### 4.2 Effects of Boron on Total Soluble Protein Content

Plants produce various osmolytes to survive ionic, oxidative, and osmotic stress. Compatible osmolytes, such as proline, GB, and sugars, prevent water loss, increase cellular turgor, and facilitate cellular expansion.^100,101^ Ancestral hulled wheat has higher soluble protein content than modern wheat.^64^ Many osmolytes synthesized under salt stress also accumulate under B and heavy metal stress, with biosynthesis being species- and tissue-specific.^101^ Exogenous osmolytes alleviate salt and metal stress.^64,102−104^ Salt-tolerant bean varieties have lower protein but higher proline and amino acid levels than salt-sensitive varieties.^105^ In our study, total soluble protein increased 16–40% in roots and 20–60% in shoots up to 15 mg L^–1^ B. GB-supplemented B increased root protein by 106% and shoot protein by 140–374% (Figure 3a,b). These increases suggest that both B and GB contribute to ROS defense in hulled wheat, potentially by stimulating enzymatic and nonenzymatic antioxidant production.

### 4.3 Effects of Boron on Antioxidant Enzyme Activity

High levels of ROS can induce oxidative damage, leading to cell death.^64^ Plants possess ROS-scavenging mechanisms. Exogenous GB alleviates oxidative stress. Calcium, zinc, GB, and silicon stimulate SOD production under stress.^83,86,106−108^ SOD catalyzes the dismutation of superoxide radicals into to H_2_O_2_ and oxygen O_2_.^109^ GB stimulates both enzymatic and nonenzymatic antioxidants, including SOD, in salt-stressed hulled wheat.^64^ H_2_O_2_ is detoxified by enzymes such as CAT and APX.^110^ Calcium increases CAT, APX, and POX activity in B-stressed radish.^107^ Silicon decreases CAT but increases APX and GP activity in rice seedlings,^111,112^ while enhancing CAT and APX activity in barley.^106^ GR maintains reduced glutathione (GSH) levels, which are essential for ROS scavenging. Calcium, silicon, and salicylic acid increase GR activity in B-stressed canola and radish.^107,113^ In our study, root SOD activity increased 4–8% with 5 mg L^–1^ B, while shoot activity increased by approximately 18% at 10 mg L^–1^ B. CAT showed the highest activity increase (176%), followed by GR (124%), GST (102%), APX (53.16%), and SOD (35.03%). The combination of B and GB increased antioxidant enzyme activities by approximately 100% (Figures 1c,d and 2c,d). Besides nutrients, certain chemicals, such as salicylic acid and GB, alleviate B toxicity.^64,114−120^ Exogenous GB doubled antioxidant enzyme activities, mitigating B-induced oxidative stress. Selenium and salicylic acid increase APX activity by upregulating ascorbate peroxidase, thereby enhancing B tolerance.^79,121−123^ APX activity increased by approximately 54% in hulled wheat treated with GB (Figure 2c,d).

**Figure 5:**
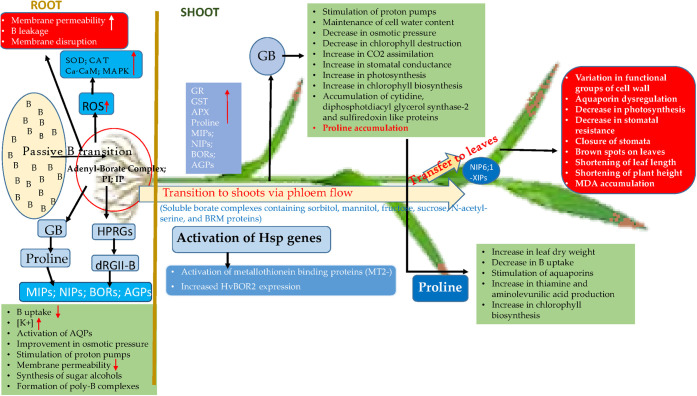
Schematic representation of the responses of hulled wheat to excessive B exposure (The mechanism is schematized in light of the information provided in Section 4.6). PI: phosphoinositide; IP: inositol phosphate; GB: Glycine betaine; MIPs: Major intrinsic proteins; MAPK: Mitogen-Activated Protein Kinases; NIPs: Nodulin26-like intrinsic proteins; BORs: Borate Exporter Family; AGPs: Arabinogalactan proteins; AQPs: Aquaporin proteins; HPRGs: Histidine/proline-hydroxyproline-rich glycoproteins; dRGII-B: Dimerized Rhamnogalacturonan II-Borate complex; -XIPs: x Intrinsic proteins; Ca-CaM: Ca^2+^-Calmodulin complex; ROS: Reactive oxygen species; SOD: Superoxide dismutase; CAT: Catalase; APX: Aspartate peroxidase; GR: Glutathione reductase; GST: Glutathione s-transferase; MDA: Malondialdehyde.

Roots, as the primary organs of contact with boron, are expected to play a leading role in the defense against B toxicity. However, unlike antioxidant defense mechanisms against other stress factors, such as salt and metal ions, the most effective defense against B stress occurs in shoots rather than roots. The main reason for this is that the plant prefers to transfer the incoming B to the shoot and remove it from the leaves, rather than preventing it from entering or neutralizing it in the roots.^45,124^ Therefore, the first toxicity symptoms encountered upon B exposure appear as brown spots on the edges of plant leaves, necrosis and chlorosis at the tips, and shortening of leaves and plant height. Subsequently, a decrease in the rate of photosynthesis due to the deterioration of the photosystem, and an increase in MDA content parallel to the increase in membrane permeability, are observed.^125,126^ However, upon initial exposure to B, wheat attempts to detoxify the initial reactive oxygen species that may occur by increasing SOD and CAT enzyme activities in the roots (Figures 5).^107,149^

### 4.4 Effect on Proline Accumulation

Plants mitigate B toxicity through mechanisms that may involve binding B to sugar alcohols. Plants synthesizing alkaloid sugars often exhibit higher tolerance to elevated B.^8,127^ Nonenzymatic antioxidants such as proline, glutathione, and aspartic acid play active roles in ROS scavenging.^128,129^ Proline levels increase under B and salt stress, and applications of plant growth regulators (PGRs) further enhance its accumulation, suggesting an adaptive effect.^64,130^ However, some studies report decreased proline levels under high B conditions, while PGRs increased it.^79,106,131,132^ Most exogenous PGRs, including GB, promote proline accumulation under high B conditions, whereas silicon inhibits proline synthesis.^131^ In our study, proline increased approximately 2-fold in roots and 4-fold in shoots due to B exposure. Exogenous GB further enhanced proline accumulation by approximately 2-fold (Tables 3 and 4; Figures 4).

### 4.5 Effect on Lipid Peroxidation (LPO) (MDA)

Excessive B is thought to induce lipid peroxidation, a key mechanism of membrane damage.^125^ Previous studies have reported increased malondialdehyde (MDA) content, indicating membrane damage with increasing B levels. Exogenous GB mitigates this damage. However, some argue against the notion that high B causes membrane damage.^133^ In our study, no significant MDA increase was observed up to 5 mg L^–1^ B in roots and 10 mg L^–1^ B in shoots. MDA levels increased 5–10 times in roots after 5 mg L^–1^ B and 1–11 times in shoots after 10 mg L^–1^ B (Tables 3 and 4; Figure 4). Exogenous GB prevented this membrane damage by approximately 80%.

### 4.6 The Role of GB and Proline in Coping with B Stress

Excess boron (B) in the soil passively enters the plant roots, increasing membrane permeability. This change in membrane permeability further increases cell membrane permeability, allowing more B to enter the cell, causing more significant damage to membranes.^77,134,135^ With B entry, the cell’s antioxidant defense, supported by SOD and CAT, is activated. This defense is accompanied by an increase in proline accumulation.^78,136^ If an osmoprotectant such as GB is present, proline accumulation increases further. The increased proline accumulation reduces B entry from the outside into the cell, increases the potassium ion concentration, and stimulates aquaporin proteins to achieve intracellular osmotic balance.^137^ As a result, intracellular osmotic balance is established, proton pumps are stimulated, membrane permeability is reduced, and the formation of sugar alcohols and poly-B complexes is promoted.^138,139^ B that enters the roots is transferred from the roots to the shoots via phloem flow.^140^ B reaching the shoots is sent toward the leaf tips and removed by guttation if conditions are suitable. If not, it accumulates in the leaf tips. B accumulated in the shoots is characterized by the formation of brown spots, especially at the leaf tips, and leads to impaired aquaporin function, decreased photosynthesis rate, shortening of leaf and plant heights, and increased MDA accumulation. To detoxify this accumulated B, the plant’s B resistance genes (Hsp genes) are activated along with the enzymatic and nonenzymatic antioxidant defense system. Hsps increase HvBOR2 expression and activate metallothionein-binding protein MT2s, thus taking a big step toward eliminating B-activated oxidative stress.^1,141−144^ Along with these, GB in the environment, as in the roots, promotes proline synthesis in the shoots, stimulates proton pumps, and helps maintain the cell water content by reducing osmotic pressure. At the same time, GB increases CO_2_ assimilation, reduces chlorophyll breakdown, and triggers an increase in the rate of photosynthesis by increasing stomatal conductance.^80^ In addition to all these, it promotes the accumulation of proteins such as cytidine diphosphate diacylglycerol synthase-2 , and sulfiredoxin.^145^ The increase in proline synthesis leads to an increase in leaf dry weight, a decrease in B uptake, the stimulation of aquaporin proteins, an increase in thiamine and aminolevulinic acid production, and ultimately an increase in chlorophyll biosynthesis^39^ (Figure 5).

### 4.7 Utilizing Ancestral Wheat for Boron Resistance

Ancestral hulled wheat varieties, in particular, are more successful in coping with abnormal growing conditions, especially biotic and abiotic stresses, compared to modern wheat varieties.^146^ This success stems from their ability to endure threats in the natural ecosystem (such as heat, cold, pathogens, nutrient deficiencies, and area competition) on their own and with their inherent abilities. For those unable to cope with these elements, natural selection leads to their extinction over time, allowing only successful species to survive. The aim of breeding programs is to identify the traits that enable these successful species to thrive, combine them in certain species, and create superior cultivars. However, these newly developed modern species are unable to adapt to changes in the evolving world as effectively as their ancestors due to the loss of genetic traits, resulting in their eventual failure.^147,148^ For this reason, there is a continuous need to scan new ancestral species in breeding programs, uncover their adaptation mechanisms to changing processes, and integrate them into breeding programs. Given boron’s critical role as an element influencing plant growth and development, encompassing both supportive and inhibitory effects, the identification and characterization of boron-resistant ancestral wheat genotypes for integration into breeding programs is of paramount importance. This is particularly relevant in regions characterized by boron-rich soils, such as Türkiye, where environmental challenges necessitate the development of tolerant cultivars.^4^

## 5 Conclusion

The detrimental effects of excessive boron on agricultural lands and ecosystems have been extensively documented. Various strategies, including nutrient supplementation, application of PGRs or plant growth-promoting microorganisms (PGPMs), liming, foliar washing, organic matter addition, and exogenous application of compounds such as proline, glycine betaine, melatonin, and silicon, have been employed to mitigate B toxicity in plants. Three primary approaches have been proposed to address B toxicity in plants: (1) developing tolerant cultivars, (2) engineering B-tolerant transgenic plants, and (3) implementing strategies to reduce B toxicity in plants. While the first strategy is time-consuming, the second is limited by regulatory constraints. Consequently, the third strategy, focusing on mitigating B toxicity, is of paramount importance. Additionally, identifying and incorporating B-tolerant ancestral species into breeding programs holds significant potential for agricultural reclamation of B-toxic lands. In this study, we evaluated the response of four ancestral hulled wheat varieties from Türkiye to high B levels in a laboratory setting, aiming to assess their suitability for breeding programs and agricultural reclamation of B-toxic soils. Despite wheat’s sensitivity to B toxicity, *T. speltoides* and *T. boeoticum* exhibited a robust antioxidant response to high B stress. These species show promise for the effective reclamation of B-contaminated agricultural areas. Furthermore, our study demonstrated the positive impact of exogenous GB application on mitigating B stress in plants. Future research is necessary to elucidate the molecular mechanisms underlying B resistance in hulled wheat.

As this study was conducted under laboratory conditions, there is a need to validate the findings under field conditions. Real-world field conditions, unlike the controlled conditions of a laboratory, involve many unpredictable positive and/or negative factors, and plants must contend with all of these conditions. Therefore, it is essential to test the findings obtained from this study under natural conditions. Testing factors such as stress tolerance under field conditions is time-consuming and requires significant financial and labor resources. Furthermore, there is no guarantee that findings obtained under laboratory conditions can be replicated in natural environments. Nevertheless, initiating long-term field studies with varieties that have shown success in laboratory settings can minimize these costs and increase the potential success rate. Considering that the majority of adverse effects caused by excess B in plants occur in shoots and leaves, the most cost-effective way to mitigate these negative impacts is to prevent B entry into plant roots. This approach minimizes B transfer from the roots to the shoots and leaves. Enhancing the regulation of B transporters and increasing the synthesis of various sugars, polyols, or thiol compounds with the potential to form complexes with B, through genetic manipulation or other molecular mechanisms, would be beneficial in inhibiting B entry into roots. Furthermore, elucidating the resistance mechanisms of B-tolerant wheat varieties such as *T. speltoides* and *T. boeoticum* through genomic and proteomic studies could provide new roadmaps for coping with B stress.

## Data Availability

All the data generated or analyzed during this study are included in this published article.

## Supporting Information Available

Table S1. Effects of borax and GB applications on the root development of hulled wheats; Table S2. Effects of borax and GB applications on the stem development of hulled wheats; Table S3. Antioxidant responses caused by borax and GB applications in the roots of hulled wheats; Table S4. Antioxidant responses caused by borax and GB applications in the stems of hulled wheats; Table S5. Tests of between-subjects effects; Table S6. Levene’s test of equality of error variances; Table S7. Multiple comparisons of hulled wheat species; Table S8. MANOVA multivariate tests (PDF)
