# Supplementary material for: Ancient Hulled Wheat: An Antioxidant-Rich Crop for Boron-Contaminated Soils
Source: ACS Omega. 2025 Apr 8;10(15):15334–50. doi: 10.1021/acsomega.4c11314 (PMC12019734; doi:10.1021/acsomega.4c11314)
Supplement: Supplementary file 1 — ao4c11314_si_001.pdf [file ao4c11314_si_001.pdf]

Ancient Hulled Wheat: An Antioxidant-Rich Crop for Boron-Contaminated Soils

Ridvan TEMIZGUL

Department of Biology, Faculty of Sciences, Erciyes University, 38039 Kayseri/TÜRKİYE

Table S1. Effects of Borax and GB applications on the root development of hulled wheats

Applications	Wheats	FW (gr)	DW (gr)	DW/FW (%)	RL(cm)
Control	Tm	4.24 ± 0.15^{d}	0.58 ± 0.03^{h}	13.68 ± 0.54^{g}	8.08 ± 0.36^{bc}
	Td	4.23 ± 0.15^{d}	0.68 ± 0.03^{g}	16.08 ± 0.64^{ef}	8.26 ± 0.37^{bc}
	Ts	4.22 ± 0.15^{d}	0.38 ± 0.02^{j}	9.01 ± 0.36^{i}	7.44 ± 0.33^{cd}
	Tb	4.28 ± 0.15^{d}	0.48 ± 0.02^{i}	11.22 ± 0.44^{h}	7.65 ± 0.34^{c}
Control + 1 mM GB	Tm	5.63 ± 0.19^{a}	0.93 ± 0.05^{d}	16.52 ± 0.66^{ef}	8.76 ± 0.39^{b}
	Td	5.54 ± 0.19^{a}	0.91 ± 0.05^{d}	16.43 ± 0.65^{ef}	8.94 ± 0.40^{b}
	Ts	5.42 ± 0.18^{b}	0.89 ± 0.05^{e}	16.42 ± 0.65^{ef}	8.63 ± 0.38^{b}
	Tb	5.86 ± 0.20^{a}	0.99 ± 0.05^{d}	16.89 ± 0.67^{ef}	9.07 ± 0.40^{ab}
1 mg L^{-1} Borax	Tm	4.36 ± 0.15^{d}	0.64 ± 0.03^{g}	14.68 ± 0.58^{fg}	8.17 ± 0.36^{bc}
	Td	4.39 ± 0.15^{d}	0.66 ± 0.03^{g}	15.03 ± 0.60^{f}	8.33 ± 0.37^{bc}
	Ts	4.37 ± 0.15^{d}	0.62 ± 0.03^{g}	14.19 ± 0.56^{fg}	7.57 ± 0.34^{c}
	Tb	4.65 ± 0.16^{c}	0.68 ± 0.03^{g}	14.62 ± 0.58^{fg}	7.81 ± 0.35^{c}
1 mg L^{-1} Borax + 1 mM GB	Tm	5.79 ± 0.20^{a}	1.14 ± 0.06^{b}	19.69 ± 0.78^{d}	9.04 ± 0.40^{ab}
	Td	5.74 ± 0.20^{a}	1.12 ± 0.06^{b}	19.51 ± 0.78^{d}	9.22 ± 0.41^{ab}
	Ts	5.71 ± 0.19^{a}	1.14 ± 0.06^{b}	19.96 ± 0.79^{d}	9.05 ± 0.40^{ab}
	Tb	5.82 ± 0.20^{a}	1.15 ± 0.06^{b}	19.76 ± 0.79^{d}	9.91 ± 0.44^{a}
5 mg L^{-1} Borax	Tm	4.54 ± 0.15^{c}	0.82 ± 0.04^{e}	18.06 ± 0.72^{de}	8.02 ± 0.36^{bc}
	Td	4.71 ± 0.16^{c}	0.84 ± 0.04^{e}	17.83 ± 0.71^{e}	8.08 ± 0.36^{bc}
	Ts	4.78 ± 0.16^{c}	0.85 ± 0.04^{e}	17.78 ± 0.71^{e}	7.24 ± 0.32^{cd}
	Tb	4.96 ± 0.17^{c}	0.88 ± 0.04^{e}	17.74 ± 0.70^{e}	7.45 ± 0.33^{cd}
5 mg L^{-1} Borax + 1 mM GB	Tm	5.89 ± 0.20^{a}	1.21 ± 0.06^{a}	20.54 ± 0.82^{cd}	8.94 ± 0.40^{b}
	Td	5.86 ± 0.20^{a}	1.26 ± 0.06^{a}	21.50 ± 0.86^{c}	9.03 ± 0.40^{ab}
	Ts	5.88 ± 0.20^{a}	1.19 ± 0.06^{b}	20.24 ± 0.80^{cd}	8.85 ± 0.39^{b}
	Tb	5.99 ± 0.20^{a}	1.28 ± 0.06^{a}	21.37 ± 0.85^{c}	9.14 ± 0.41^{ab}
10 mg L^{-1} Borax	Tm	4.22 ± 0.14^{d}	0.78 ± 0.04^{f}	18.48 ± 0.73^{de}	7.63 ± 0.34^{c}
	Td	4.18 ± 0.14^{d}	0.78 ± 0.04^{f}	18.66 ± 0.74^{de}	7.65 ± 0.34^{c}
	Ts	4.25 ± 0.14^{d}	0.81 ± 0.04^{e}	19.06 ± 0.76^{d}	7.02 ± 0.31^{cd}
	Tb	4.32 ± 0.15^{d}	0.84 ± 0.04^{e}	19.44 ± 0.77^{d}	7.07 ± 0.31^{cd}
10 mg L^{-1} Borax + 1 mM GB	Tm	5.01 ± 0.17^{b}	1.18 ± 0.06^{b}	23.55 ± 0.92^{b}	8.13 ± 0.36^{bc}
	Td	5.04 ± 0.17^{b}	1.16 ± 0.06^{b}	23.02 ± 0.92^{b}	8.18 ± 0.36^{bc}
	Ts	4.89 ± 0.17^{c}	1.26 ± 0.06^{a}	25.77 ± 1.03^{a}	7.96 ± 0.35^{c}
	Tb	5.16 ± 0.18^{b}	1.28 ± 0.06^{a}	24.81 ± 0.99^{ab}	8.45 ± 0.38^{bc}
15 mg L^{-1} Borax	Tm	4.03 ± 0.14^{d}	0.74 ± 0.04^{f}	18.36 ± 0.73^{de}	7.41 ± 0.33^{cd}
	Td	3.96 ± 0.13^{e}	0.75 ± 0.03^{f}	18.94 ± 0.75^{de}	7.36 ± 0.33^{cd}
	Ts	4.06 ± 0.14^{d}	0.78 ± 0.04^{f}	19.21 ± 0.76^{d}	6.73 ± 0.30^{d}
	Tb	4.18 ± 0.14^{d}	0.79 ± 0.04^{f}	18.90 ± 0.75^{de}	6.77 ± 0.30^{d}
15 mg L^{-1} Borax + 1 mM GB	Tm	4.71 ± 0.16^{c}	1.12 ± 0.05^{b}	23.78 ± 0.95^{b}	7.78 ± 0.35^{c}
	Td	4.74 ± 0.16^{c}	1.10 ± 0.05^{b}	23.21 ± 0.92^{b}	7.76 ± 0.34^{c}
	Ts	4.75 ± 0.16^{c}	1.13 ± 0.05^{b}	23.79 ± 0.95^{b}	7.15 ± 0.32^{cd}
	Tb	4.88 ± 0.17^{c}	1.16 ± 0.06^{b}	23.77 ± 0.95^{b}	7.21 ± 0.32^{cd}
20 mg L^{-1} Borax	Tm	3.44 ± 0.12^{f}	0.68 ± 0.03^{g}	19.77 ± 0.79^{d}	6.88 ± 0.30^{d}
	Td	3.38 ± 0.11^{f}	0.65 ± 0.03^{g}	19.23 ± 0.76^{d}	6.74 ± 0.30^{d}
	Ts	3.41 ± 0.11^{f}	0.69 ± 0.03^{g}	20.23 ± 0.80^{cd}	6.91 ± 0.31^{d}
	Tb	3.46 ± 0.12^{f}	0.72 ± 0.04^{f}	20.81 ± 0.83^{cd}	6.94 ± 0.31^{d}
20 mg L^{-1} Borax + 1 mM GB	Tm	3.91 ± 0.13^{e}	0.82 ± 0.04^{e}	20.97 ± 0.83^{cd}	7.24 ± 0.32^{cd}
	Td	3.94 ± 0.13^{e}	0.81 ± 0.04^{e}	20.56 ± 0.82^{cd}	7.19 ± 0.32^{cd}
	Ts	3.92 ± 0.13^{e}	0.83 ± 0.04^{e}	21.17 ± 0.84^{c}	7.25 ± 0.32^{cd}
	Tb	3.98 ± 0.13^{e}	0.86 ± 0.04^{e}	21.61 ± 0.86^{c}	7.23 ± 0.32^{cd}

FW: Fresh weight; DW: Dry weight; RL: Root length; GB: Glycine-Betaine; Tm: *T. monococcum*; Td: *T. dicoccum*; Ts: *T. speltoides*; Tb: *T. boeoticum*; *Differences in the letters indicates statistical significance at 5% level in the columns.

Table S2. Effects of Borax and GB applications on the stem development of hulled wheats

Applications	Wheats	FW (gr)	DW (gr)	DW/FW (%)	PH (cm)	LL (cm)	Chl_a (mg gr^{-1} fw)	Chl_b (mg gr^{-1} fw)	Chl a/b	Total Chl (mg gr^{-1} fw)	Carotene (mg gr^{-1} fw)
Control	Tm	28.28 ± 1.27[gh]	3.79 ± 0.17[de]	13.40 ± 0.60[cd]	31.59 ± 1.42[d]	18.67 ± 0.84[f]	1.81 ± 0.08[cd]	0.78 ± 0.03[d]	2.32 ± 0.10[b]	2.59 ± 0.11[fg]	0.51 ± 0.02[d]
	Td	27.22 ± 1.22[h]	3.98 ± 0.17[de]	14.62 ± 0.65[c]	35.11 ± 1.57[c]	20.54 ± 0.92[e]	1.90 ± 0.08[c]	0.81 ± 0.03[cd]	2.35 ± 0.10[b]	2.71 ± 0.12[ef]	0.40 ± 0.01[e]
	Ts	30.98 ± 1.39[fg]	3.21 ± 0.14[ef]	10.36 ± 0.46[e]	25.74 ± 1.15[ef]	16.80 ± 0.75[g]	1.71 ± 0.07[d]	0.80 ± 0.03[cd]	2.14 ± 0.09[c]	2.51 ± 0.11[fg]	0.62 ± 0.02[cd]
	Tb	31.36 ± 1.41[f]	3.40 ± 0.15[e]	10.84 ± 0.48[e]	28.08 ± 1.26[de]	17.73 ± 0.79[fg]	1.67 ± 0.07[de]	0.81 ± 0.03[cd]	2.06 ± 0.09[cd]	2.48 ± 0.11[g]	0.59 ± 0.02[d]
Control + 1 mM GB	Tm	30.44 ± 1.36[fg]	4.56 ± 0.20[cd]	14.98 ± 0.67[bc]	36.65 ± 1.64[bc]	22.54 ± 1.01[d]	2.01 ± 0.09[bc]	0.91 ± 0.04[c]	2.21 ± 0.09[bc]	2.92 ± 0.13[de]	0.54 ± 0.02[d]
	Td	30.02 ± 1.35[fg]	4.51 ± 0.20[cd]	15.02 ± 0.67[bc]	39.32 ± 1.76[b]	23.46 ± 1.05[cd]	2.05 ± 0.09[bc]	0.92 ± 0.04[c]	2.23 ± 0.10[bc]	2.97 ± 0.13[de]	0.45 ± 0.02[e]
	Ts	34.56 ± 1.55[de]	4.59 ± 0.20[cd]	13.28 ± 0.59[cd]	34.87 ± 1.56[c]	24.67 ± 1.11[c]	1.92 ± 0.08[c]	0.88 ± 0.03[cd]	2.18 ± 0.09[c]	2.80 ± 0.12[e]	0.68 ± 0.03[cd]
	Tb	36.22 ± 1.62[cd]	4.63 ± 0.20[cd]	12.78 ± 0.57[d]	38.54 ± 1.73[b]	26.08 ± 1.17[b]	1.90 ± 0.08[c]	0.89 ± 0.04[cd]	2.13 ± 0.09[c]	2.79 ± 0.12[ef]	0.65 ± 0.02[cd]
1 mg L^{-1} Borax	Tm	29.42 ± 1.32[g]	4.50 ± 0.20[cd]	15.29 ± 0.68[bc]	33.24 ± 1.49[cd]	19.74 ± 0.88[ef]	2.11 ± 0.09[b]	0.97 ± 0.04[c]	2.18 ± 0.09[c]	3.08 ± 0.13[d]	0.57 ± 0.02[d]
	Td	29.14 ± 1.31[g]	4.48 ± 0.20[cd]	15.37 ± 0.69[bc]	38.62 ± 1.73[b]	21.78 ± 0.98[de]	2.18 ± 0.09[b]	0.97 ± 0.04[c]	2.25 ± 0.10[bc]	3.15 ± 0.14[cd]	0.48 ± 0.02[e]
	Ts	32.26 ± 1.45[ef]	4.54 ± 0.20[cd]	14.07 ± 0.63[c]	31.86 ± 1.43[d]	21.57 ± 0.97[de]	2.02 ± 0.09[bc]	0.96 ± 0.04[c]	2.10 ± 0.09[c]	2.98 ± 0.13[de]	0.71 ± 0.03[c]
	Tb	32.98 ± 1.48[ef]	4.56 ± 0.20[cd]	13.82 ± 0.62[cd]	36.64 ± 1.64[bc]	24.88 ± 1.11[c]	1.99 ± 0.08[c]	0.95 ± 0.04[c]	2.09 ± 0.09[cd]	2.94 ± 0.13[de]	0.70 ± 0.03[c]
1 mg L^{-1} Borax + 1 mM GB	Tm	31.63 ± 1.42[f]	5.07 ± 0.22[bc]	16.02 ± 0.72[b]	39.08 ± 1.75[b]	23.36 ± 1.05[cd]	2.18 ± 0.09[b]	1.05 ± 0.04[bc]	2.08 ± 0.09[cd]	3.23 ± 0.14[c]	0.65 ± 0.02[cd]
	Td	31.45 ± 1.41[f]	5.01 ± 0.22[bc]	15.93 ± 0.71[bc]	41.25 ± 1.85[ab]	24.75 ± 1.11[c]	2.31 ± 0.10[a]	1.07 ± 0.04[bc]	2.16 ± 0.09[c]	3.38 ± 0.15[bc]	0.54 ± 0.02[d]
	Ts	36.08 ± 1.62[cd]	5.09 ± 0.22[bc]	14.10 ± 0.63[c]	38.56 ± 1.73[b]	26.21 ± 1.17[b]	2.16 ± 0.09[b]	1.04 ± 0.04[bc]	2.08 ± 0.09[cd]	3.20 ± 0.14[c]	0.79 ± 0.03[c]
	Tb	36.94 ± 1.66[cd]	5.12 ± 0.23[bc]	13.86 ± 0.62[cd]	40.25 ± 1.81[ab]	28.44 ± 1.27[a]	2.17 ± 0.09[b]	1.05 ± 0.04[bc]	2.07 ± 0.09[cd]	3.22 ± 0.14[c]	0.77 ± 0.03[c]
5 mg L^{-1} Borax	Tm	30.65 ± 1.37[fg]	4.89 ± 0.22[c]	15.95 ± 0.71[bc]	33.36 ± 1.50[cd]	19.72 ± 0.88[ef]	2.14 ± 0.09[b]	1.07 ± 0.04[bc]	2.00 ± 0.09[cd]	3.21 ± 0.14[c]	0.62 ± 0.02[cd]
	Td	30.68 ± 1.38[fg]	4.91 ± 0.22[c]	16.00 ± 0.72[b]	38.78 ± 1.74[b]	21.82 ± 0.98[de]	2.23 ± 0.10[ab]	1.08 ± 0.04[bc]	2.06 ± 0.09[cd]	3.31 ± 0.14[bc]	0.54 ± 0.02[d]
	Ts	33.72 ± 1.51[e]	4.94 ± 0.22[c]	14.65 ± 0.65[c]	32.04 ± 1.44[cd]	21.34 ± 0.96[de]	2.11 ± 0.09[b]	1.07 ± 0.04[bc]	1.97 ± 0.08[d]	3.18 ± 0.14[cd]	0.75 ± 0.03[c]
	Tb	33.85 ± 1.52[e]	4.97 ± 0.22[c]	14.68 ± 0.66[c]	36.94 ± 1.66[bc]	24.51 ± 1.10[c]	2.08 ± 0.09[bc]	1.06 ± 0.04[bc]	1.96 ± 0.08[d]	3.14 ± 0.14[cd]	0.79 ± 0.03[c]
5 mg L^{-1} Borax + 1 mM GB	Tm	33.98 ± 1.52[e]	5.66 ± 0.25[b]	16.65 ± 0.74[b]	41.25 ± 1.85[ab]	23.78 ± 1.07[cd]	2.21 ± 0.09[ab]	1.22 ± 0.05[ab]	1.81 ± 0.08[de]	3.43 ± 0.15[b]	0.75 ± 0.03[c]
	Td	33.94 ± 1.52[e]	5.61 ± 0.25[b]	16.52 ± 0.74[b]	42.78 ± 1.92[a]	24.96 ± 1.12[c]	2.38 ± 0.10[a]	1.23 ± 0.05[ab]	1.93 ± 0.08[d]	3.61 ± 0.16[a]	0.66 ± 0.02[cd]
	Ts	38.17 ± 1.71[bc]	5.67 ± 0.25[b]	14.85 ± 0.66[c]	42.12 ± 1.89[a]	26.65 ± 1.19[b]	2.33 ± 0.10[a]	1.23 ± 0.05[ab]	1.89 ± 0.08[de]	3.56 ± 0.16[ab]	0.90 ± 0.04[b]
	Tb	38.55 ± 1.73[bc]	5.69 ± 0.25[ab]	14.76 ± 0.66[c]	43.46 ± 1.95[a]	28.98 ± 1.30[a]	2.36 ± 0.10[a]	1.24 ± 0.05[ab]	1.90 ± 0.08[d]	3.60 ± 0.16[a]	0.91 ± 0.04[b]
10 mg L^{-1} Borax	Tm	30.44 ± 1.36[fg]	5.01 ± 0.22[c]	16.45 ± 0.74[b]	30.72 ± 1.38[d]	18.21 ± 0.81[f]	2.01 ± 0.09[bc]	1.05 ± 0.04[bc]	1.91 ± 0.08[d]	3.06 ± 0.13[d]	0.67 ± 0.03[cd]
	Td	30.56 ± 1.37[fg]	4.98 ± 0.22[c]	16.29 ± 0.73[b]	34.44 ± 1.54[c]	20.14 ± 0.90[e]	2.03 ± 0.09[bc]	1.04 ± 0.04[bc]	1.95 ± 0.08[d]	3.07 ± 0.13[d]	0.60 ± 0.02[cd]
	Ts	34.08 ± 1.53[de]	5.02 ± 0.22[c]	14.73 ± 0.66[c]	24.91 ± 1.12[ef]	20.26 ± 0.91[e]	1.92 ± 0.08[c]	1.02 ± 0.04[bc]	1.88 ± 0.08[de]	2.94 ± 0.13[de]	0.82 ± 0.03[bc]
	Tb	34.25 ± 1.54[de]	5.05 ± 0.22[bc]	14.74 ± 0.66[c]	27.16 ± 1.22[e]	22.84 ± 1.02[d]	1.94 ± 0.08[c]	1.03 ± 0.04[bc]	1.88 ± 0.08[de]	2.97 ± 0.13[de]	0.91 ± 0.04[b]
10 mg L^{-1} Borax + 1 mM GB	Tm	35.02 ± 1.57[d]	6.04 ± 0.27[a]	17.24 ± 0.77[ab]	35.08 ± 1.57[c]	22.12 ± 0.99[d]	2.09 ± 0.09[bc]	1.30 ± 0.05[a]	1.61 ± 0.07[ef]	3.39 ± 0.15[bc]	0.84 ± 0.03[bc]
	Td	35.54 ± 1.59[d]	6.01 ± 0.27[a]	16.91 ± 0.76[b]	38.24 ± 1.72[b]	22.28 ± 1.00[d]	2.12 ± 0.09[b]	1.31 ± 0.05[a]	1.62 ± 0.07[ef]	3.43 ± 0.15[b]	0.80 ± 0.03[bc]
	Ts	41.78 ± 1.88[a]	6.07 ± 0.27[a]	14.52 ± 0.65[c]	32.96 ± 1.48[cd]	23.81 ± 1.07[cd]	2.02 ± 0.09[bc]	1.28 ± 0.05[ab]	1.58 ± 0.07[f]	3.30 ± 0.14[bc]	1.02 ± 0.04[ab]
	Tb	41.91 ± 1.88[a]	6.11 ± 0.27[a]	14.57 ± 0.65[c]	36.41 ± 1.63[bc]	24.87 ± 1.11[c]	2.05 ± 0.09[bc]	1.27 ± 0.05[ab]	1.61 ± 0.07[ef]	3.32 ± 0.14[bc]	1.04 ± 0.04[ab]
15 mg L^{-1} Borax	Tm	28.12 ± 1.26[gh]	4.75 ± 0.21[c]	16.89 ± 0.76[b]	28.02 ± 1.26[de]	16.96 ± 0.76[g]	1.78 ± 0.08[d]	0.89 ± 0.04[cd]	2.00 ± 0.09[cd]	2.67 ± 0.12[f]	0.65 ± 0.02[cd]
	Td	27.98 ± 1.25[h]	4.72 ± 0.21[c]	16.86 ± 0.75[b]	31.63 ± 1.42[d]	18.74 ± 0.84[f]	1.75 ± 0.07[d]	0.88 ± 0.03[cd]	1.99 ± 0.08[d]	2.63 ± 0.11[f]	0.59 ± 0.02[d]
	Ts	30.54 ± 1.37[fg]	4.76 ± 0.21[c]	15.58 ± 0.70[bc]	23.76 ± 1.06[f]	18.85 ± 0.84[f]	1.64 ± 0.07[de]	0.87 ± 0.03[cd]	1.89 ± 0.08[de]	2.51 ± 0.11[fg]	0.80 ± 0.03[bc]
	Tb	30.38 ± 1.36[fg]	4.79 ± 0.21[c]	15.76 ± 0.70[bc]	25.74 ± 1.15[ef]	19.98 ± 0.89[ef]	1.65 ± 0.07[de]	0.88 ± 0.03[cd]	1.88 ± 0.08[de]	2.53 ± 0.11[fg]	0.86 ± 0.03[bc]
15 mg L^{-1} Borax + 1 mM GB	Tm	31.44 ± 1.41[f]	5.60 ± 0.25[b]	17.81 ± 0.80[ab]	34.36 ± 1.54[c]	21.63 ± 0.97[de]	1.92 ± 0.08[c]	1.11 ± 0.04[b]	1.73 ± 0.07[e]	3.03 ± 0.13[d]	0.85 ± 0.03[bc]
	Td	31.57 ± 1.42[f]	5.54 ± 0.24[b]	17.54 ± 0.78[ab]	36.55 ± 1.64[bc]	22.01 ± 0.99[d]	1.93 ± 0.08[c]	1.09 ± 0.04[bc]	1.77 ± 0.07[e]	3.02 ± 0.13[d]	0.82 ± 0.03[bc]
	Ts	37.76 ± 1.69[c]	5.63 ± 0.25[b]	14.90 ± 0.67[c]	32.02 ± 1.44[cd]	22.24 ± 1.00[d]	1.90 ± 0.08[c]	1.10 ± 0.04[bc]	1.73 ± 0.07[e]	3.00 ± 0.13[d]	1.06 ± 0.04[ab]
	Tb	37.68 ± 1.69[c]	5.67 ± 0.25[ab]	15.04 ± 0.67[bc]	35.14 ± 1.58[c]	23.11 ± 1.03[cd]	1.91 ± 0.08[c]	1.12 ± 0.05[b]	1.71 ± 0.07[e]	3.03 ± 0.13[d]	1.10 ± 0.04[a]
20 mg L^{-1} Borax	Tm	26.13 ± 1.17[hi]	4.52 ± 0.20[cd]	17.29 ± 0.77[ab]	26.08 ± 1.17[e]	16.14 ± 0.72[g]	1.54 ± 0.06[e]	0.62 ± 0.02[e]	2.48 ± 0.11[ab]	2.16 ± 0.09[i]	0.58 ± 0.02[d]
	Td	26.02 ± 1.17[hi]	4.47 ± 0.20[cd]	17.17 ± 0.77[ab]	27.34 ± 1.23[e]	17.21 ± 0.77[fg]	1.51 ± 0.06[e]	0.62 ± 0.02[e]	2.44 ± 0.10[ab]	2.13 ± 0.09[i]	0.57 ± 0.02[d]
	Ts	26.74 ± 1.20[hi]	4.51 ± 0.20[cd]	16.86 ± 0.75[b]	23.15 ± 1.04[f]	17.25 ± 0.77[fg]	1.56 ± 0.07[e]	0.63 ± 0.02[e]	2.48 ± 0.11[ab]	2.19 ± 0.09[i]	0.78 ± 0.03[c]
	Tb	26.91 ± 1.21[hi]	4.56 ± 0.20[cd]	16.94 ± 0.76[b]	25.56 ± 1.15[ef]	17.46 ± 0.78[fg]	1.58 ± 0.07[e]	0.63 ± 0.02[e]	2.51 ± 0.11[a]	2.21 ± 0.09[h]	0.83 ± 0.03[bc]
20 mg L^{-1} Borax + 1 mM GB	Tm	28.66 ± 1.28[gh]	5.22 ± 0.23[bc]	18.21 ± 0.81[a]	32.75 ± 1.47[cd]	20.18 ± 0.90[e]	1.72 ± 0.07[d]	0.77 ± 0.03[d]	2.23 ± 0.10[bc]	2.49 ± 0.11[g]	0.88 ± 0.03[bc]
	Td	28.03 ± 1.26[gh]	5.19 ± 0.23[bc]	18.51 ± 0.83[a]	35.88 ± 1.61[c]	20.75 ± 0.93[e]	1.70 ± 0.07[d]	0.78 ± 0.03[d]	2.18 ± 0.09[c]	2.48 ± 0.11[g]	0.85 ± 0.03[bc]
	Ts	31.47 ± 1.41[f]	5.23 ± 0.23[bc]	16.61 ± 0.74[b]	30.16 ± 1.35[d]	20.79 ± 0.93[e]	1.75 ± 0.07[d]	0.78 ± 0.03[d]	2.24 ± 0.10[bc]	2.53 ± 0.11[fg]	1.04 ± 0.04[ab]
	Tb	32.65 ± 1.46[ef]	5.28 ± 0.23[b]	16.17 ± 0.72[b]	33.72 ± 1.51[cd]	20.85 ± 0.93[e]	1.79 ± 0.08[d]	0.79 ± 0.03[d]	2.27 ± 0.10[bc]	2.58 ± 0.11[fg]	1.12 ± 0.05[a]

FW: Fresh weight; DW: Dry weight; PH: Plant height; LL: Leaf length; GB: Glycine-Betaine; *Differences in the letters indicates statistical significance at 5% level in the columns.

Table S3. Antioxidant responses caused by Borax and GB applications in the root of hulled wheats

Applications	Wheats	TPC (µg ml^{-1} protein)	SOD (U ml^{-1} protein)	CAT (U ml^{-1} protein)	GR (U ml^{-1} protein)	GST (U ml^{-1} protein)	APX (U ml^{-1} protein)	PRO (nmol gr^{-1} fw)	MDA (nmol gr^{-1} fw)
Control	Tm	500.05 ± 22.50^{gh}	1.118 ± 0.05^{ef}	0.034 ± 0.001^{e}	0.117 ± 0.005^{d}	0.087 ± 0.004^{e}	0.538 ± 0.024^{de}	23.55 ± 1.06^{e}	7.35 ± 0.33^{e}
	Td	548.47 ± 24.68^{g}	1.172 ± 0.05^{de}	0.017 ± 0.001^{h}	0.103 ± 0.005^{d}	0.087 ± 0.004^{e}	0.478 ± 0.022^{e}	26.50 ± 1.19^{e}	5.99 ± 0.27^{e}
	Ts	415.32 ± 18.68^{i}	1.254 ± 0.05^{cd}	0.035 ± 0.001^{de}	0.176 ± 0.008^{c}	0.173 ± 0.008^{cd}	0.637 ± 0.029^{cd}	36.75 ± 1.65^{de}	8.24 ± 0.37^{e}
	Tb	449.90 ± 20.24^{hi}	1.207 ± 0.05^{d}	0.035 ± 0.001^{de}	0.176 ± 0.008^{c}	0.161 ± 0.007^{cd}	0.558 ± 0.025^{d}	31.63 ± 1.42^{e}	7.49 ± 0.34^{e}
Control + 1 mM GB	Tm	675.28 ± 30.38^{e}	1.196 ± 0.05^{de}	0.036 ± 0.002^{de}	0.132 ± 0.006^{cd}	0.098 ± 0.004^{de}	0.574 ± 0.026^{d}	32.70 ± 1.47^{e}	6.64 ± 0.30^{e}
	Td	705.26 ± 31.73^{de}	1.208 ± 0.05^{d}	0.021 ± 0.001^{g}	0.138 ± 0.006^{cd}	0.096 ± 0.004^{de}	0.561 ± 0.025^{d}	32.88 ± 1.48^{e}	5.90 ± 0.27^{e}
	Ts	623.98 ± 28.07^{ef}	1.344 ± 0.06^{b}	0.039 ± 0.002^{d}	0.218 ± 0.010^{bc}	0.203 ± 0.009^{c}	0.705 ± 0.032^{bc}	41.13 ± 1.85^{d}	6.42 ± 0.29^{e}
	Tb	626.28 ± 28.18^{ef}	1.278 ± 0.05^{c}	0.040 ± 0.002^{d}	0.233 ± 0.010^{bc}	0.201 ± 0.009^{c}	0.686 ± 0.031^{c}	38.41 ± 1.72^{de}	6.48 ± 0.29^{e}
1 mg L^{-1} Borax	Tm	565.76 ± 25.45^{fg}	1.146 ± 0.05^{e}	0.037 ± 0.002^{de}	0.139 ± 0.006^{cd}	0.092 ± 0.004^{de}	0.561 ± 0.025^{d}	25.42 ± 1.14^{e}	8.02 ± 0.36^{e}
	Td	594.01 ± 26.73^{f}	1.196 ± 0.05^{de}	0.020 ± 0.001^{gh}	0.133 ± 0.006^{cd}	0.091 ± 0.004^{de}	0.514 ± 0.023^{de}	29.38 ± 1.32^{e}	7.24 ± 0.33^{e}
	Ts	441.26 ± 19.85^{hi}	1.278 ± 0.05^{c}	0.038 ± 0.002^{d}	0.218 ± 0.010^{bc}	0.194 ± 0.009^{c}	0.695 ± 0.031^{c}	41.42 ± 1.86^{d}	10.05 ± 0.45^{de}
	Tb	492.56 ± 22.16^{h}	1.234 ± 0.05^{cd}	0.039 ± 0.002^{d}	0.215 ± 0.010^{bc}	0.173 ± 0.008^{cd}	0.602 ± 0.027^{cd}	34.76 ± 1.56^{de}	9.16 ± 0.41^{e}
1 mg L^{-1} Borax + 1 mM GB	Tm	675.29 ± 30.38^{e}	1.242 ± 0.05^{cd}	0.040 ± 0.002^{d}	0.141 ± 0.006^{cd}	0.102 ± 0.005^{de}	0.604 ± 0.027^{cd}	36.45 ± 1.65^{de}	7.08 ± 0.32^{e}
	Td	705.25 ± 31.73^{de}	1.254 ± 0.05^{cd}	0.024 ± 0.001^{fg}	0.149 ± 0.007^{cd}	0.103 ± 0.005^{de}	0.592 ± 0.027^{d}	36.64 ± 1.64^{de}	6.75 ± 0.30^{e}
	Ts	623.98 ± 28.07^{ef}	1.378 ± 0.06^{ab}	0.042 ± 0.002^{cd}	0.244 ± 0.011^{b}	0.225 ± 0.010^{bc}	0.772 ± 0.035^{b}	45.76 ± 2.05^{d}	7.14 ± 0.32^{e}
	Tb	626.28 ± 28.18^{ef}	1.309 ± 0.05^{bc}	0.042 ± 0.002^{cd}	0.268 ± 0.012^{b}	0.221 ± 0.010^{bc}	0.734 ± 0.033^{bc}	44.08 ± 1.98^{d}	7.22 ± 0.32^{e}
5 mg L^{-1} Borax	Tm	619.94 ± 27.89^{f}	1.174 ± 0.05^{de}	0.042 ± 0.002^{cd}	0.154 ± 0.007^{cd}	0.104 ± 0.005^{de}	0.598 ± 0.027^{cd}	44.71 ± 2.01^{d}	10.34 ± 0.47^{de}
	Td	613.61 ± 27.61^{f}	1.236 ± 0.05^{cd}	0.029 ± 0.001^{f}	0.149 ± 0.007^{cd}	0.102 ± 0.005^{de}	0.563 ± 0.025^{d}	51.16 ± 2.30^{d}	10.46 ± 0.47^{de}
	Ts	513.88 ± 23.12^{gh}	1.305 ± 0.05^{bc}	0.047 ± 0.002^{bc}	0.263 ± 0.012^{b}	0.234 ± 0.011^{bc}	0.777 ± 0.035^{b}	63.88 ± 2.87^{cd}	14.79 ± 0.67^{de}
	Tb	525.42 ± 23.64^{gh}	1.297 ± 0.05^{bc}	0.051 ± 0.002^{b}	0.265 ± 0.012^{b}	0.218 ± 0.010^{bc}	0.741 ± 0.033^{bc}	55.48 ± 2.48^{d}	13.85 ± 0.62^{de}
5 mg L^{-1} Borax + 1 mM GB	Tm	721.39 ± 32.46^{d}	1.283 ± 0.05^{c}	0.046 ± 0.002^{c}	0.176 ± 0.008^{c}	0.124 ± 0.006^{d}	0.645 ± 0.029^{cd}	66.72 ± 3.00^{cd}	8.46 ± 0.38^{e}
	Td	745.61 ± 33.55^{cd}	1.275 ± 0.05^{c}	0.031 ± 0.001^{ef}	0.179 ± 0.008^{c}	0.128 ± 0.006^{d}	0.619 ± 0.028^{cd}	74.81 ± 3.36^{cd}	7.33 ± 0.33^{e}
	Ts	730.05 ± 32.85^{d}	1.394 ± 0.06^{a}	0.054 ± 0.002^{ab}	0.298 ± 0.013^{ab}	0.271 ± 0.012^{ab}	0.835 ± 0.038^{ab}	83.85 ± 3.77^{c}	7.88 ± 0.35^{e}
	Tb	726.58 ± 32.69^{d}	1.346 ± 0.06^{b}	0.055 ± 0.002^{ab}	0.276 ± 0.012^{b}	0.248 ± 0.011^{b}	0.807 ± 0.036^{ab}	81.39 ± 3.66^{c}	8.01 ± 0.36^{e}
10 mg L^{-1} Borax	Tm	653.96 ± 29.42^{e}	1.102 ± 0.04^{f}	0.040 ± 0.002^{d}	0.136 ± 0.006^{cd}	0.098 ± 0.004^{de}	0.513 ± 0.023^{de}	48.60 ± 2.18^{d}	16.71 ± 0.75^{d}
	Td	627.44 ± 28.23^{ef}	1.202 ± 0.05^{d}	0.028 ± 0.001^{f}	0.131 ± 0.006^{cd}	0.097 ± 0.004^{de}	0.484 ± 0.022^{e}	55.02 ± 2.47^{d}	16.24 ± 0.73^{d}
	Ts	561.15 ± 25.25^{fg}	1.264 ± 0.05^{c}	0.044 ± 0.002^{c}	0.214 ± 0.010^{bc}	0.176 ± 0.008^{cd}	0.592 ± 0.027^{d}	71.24 ± 3.20^{cd}	24.45 ± 1.10^{cd}
	Tb	568.64 ± 25.58^{fg}	1.251 ± 0.05^{cd}	0.045 ± 0.002^{c}	0.203 ± 0.009^{bc}	0.171 ± 0.008^{cd}	0.574 ± 0.026^{d}	66.48 ± 2.99^{cd}	22.38 ± 1.01^{cd}
10 mg L^{-1} Borax + 1 mM GB	Tm	735.81 ± 33.11^{d}	1.285 ± 0.05^{c}	0.047 ± 0.002^{bc}	0.188 ± 0.008^{c}	0.147 ± 0.007^{d}	0.664 ± 0.030^{c}	105.42 ± 4.74^{bc}	12.33 ± 0.55^{de}
	Td	769.23 ± 34.61^{cd}	1.297 ± 0.05^{bc}	0.034 ± 0.001^{e}	0.205 ± 0.009^{bc}	0.156 ± 0.007^{cd}	0.651 ± 0.029^{c}	124.58 ± 5.60^{b}	12.38 ± 0.56^{de}
	Ts	700.06 ± 31.50^{de}	1.401 ± 0.06^{a}	0.057 ± 0.003^{a}	0.336 ± 0.015^{a}	0.321 ± 0.014^{a}	0.879 ± 0.040^{a}	139.31 ± 6.26^{ab}	14.74 ± 0.66d^{e}
	Tb	803.26 ± 36.14^{bc}	1.389 ± 0.06^{a}	0.056 ± 0.003^{a}	0.309 ± 0.014^{ab}	0.308 ± 0.014^{a}	0.895 ± 0.040^{a}	135.45 ± 6.09^{b}	14.11 ± 0.63^{de}
15 mg L^{-1} Borax	Tm	664.90 ± 29.92^{e}	1.024 ± 0.04^{g}	0.035 ± 0.002^{de}	0.102 ± 0.005^{d}	0.076 ± 0.003^{e}	0.425 ± 0.019^{ef}	61.03 ± 2.74^{cd}	35.63 ± 1.60^{bc}
	Td	638.96 ± 28.75^{ef}	1.163 ± 0.05^{e}	0.026 ± 0.001^{f}	0.098 ± 0.004^{d}	0.077 ± 0.003^{e}	0.401 ± 0.018^{ef}	76.40 ± 3.43^{cd}	36.09 ± 1.62^{bc}
	Ts	578.45 ± 26.03^{fg}	1.177 ± 0.05^{de}	0.036 ± 0.002^{de}	0.163 ± 0.007^{c}	0.124 ± 0.006^{d}	0.454 ± 0.020^{e}	84.35 ± 3.79^{c}	45.77 ± 2.06^{b}
	Tb	558.27 ± 25.12^{g}	1.171 ± 0.05^{de}	0.036 ± 0.002^{de}	0.155 ± 0.007^{cd}	0.128 ± 0.006^{d}	0.448 ± 0.020^{ef}	78.56 ± 3.53^{c}	45.13 ± 2.03^{b}
15 mg L^{-1} Borax + 1 mM GB	Tm	787.68 ± 35.44^{c}	1.143 ± 0.05^{e}	0.046 ± 0.002^{c}	0.184 ± 0.008^{c}	0.144 ± 0.006^{d}	0.644 ± 0.029^{cd}	112.54 ± 5.06^{bc}	13.36 ± 0.60^{de}
	Td	891.43 ± 40.11^{a}	1.208 ± 0.05^{d}	0.035 ± 0.001^{de}	0.196 ± 0.009^{c}	0.155 ± 0.007^{cd}	0.633 ± 0.028^{cd}	128.06 ± 5.76^{b}	13.68 ± 0.62^{de}
	Ts	858.01 ± 38.61^{ab}	1.314 ± 0.05^{bc}	0.055 ± 0.003^{ab}	0.311 ± 0.014^{ab}	0.314 ± 0.014^{a}	0.799 ± 0.036^{ab}	163.84 ± 7.37^{a}	16.28 ± 0.73^{d}
	Tb	837.25 ± 37.67^{b}	1.285 ± 0.05^{c}	0.054 ± 0.002^{ab}	0.295 ± 0.013^{ab}	0.302 ± 0.014^{a}	0.818 ± 0.037^{ab}	160.48 ± 7.22^{a}	15.84 ± 0.71^{d}
20 mg L^{-1} Borax	Tm	545.59 ± 24.55^{g}	0.844 ± 0.03^{j}	0.028 ± 0.001^{f}	0.081 ± 0.004^{d}	0.065 ± 0.003^{e}	0.336 ± 0.015^{g}	74.55 ± 3.35^{cd}	65.46 ± 2.95^{ab}
	Td	537.52 ± 24.18^{g}	1.015 ± 0.04^{gh}	0.021 ± 0.001^{g}	0.080 ± 0.004^{d}	0.065 ± 0.003^{e}	0.313 ± 0.014^{g}	80.42 ± 3.61^{c}	68.73 ± 3.09^{ab}
	Ts	448.17 ± 20.16^{hi}	1.022 ± 0.04^{g}	0.028 ± 0.001^{f}	0.105 ± 0.005^{d}	0.078 ± 0.004^{e}	0.325 ± 0.015^{g}	92.36 ± 4.15^{c}	75.44 ± 3.39^{a}
	Tb	457.40 ± 20.58^{hi}	1.040 ± 0.04^{g}	0.029 ± 0.001^{f}	0.102 ± 0.005^{d}	0.080 ± 0.004^{e}	0.333 ± 0.015^{g}	88.24 ± 3.97^{c}	76.62 ± 3.45^{a}
20 mg L^{-1} Borax + 1 mM GB	Tm	727.16 ± 32.72^{d}	1.018 ± 0.04^{gh}	0.041 ± 0.002^{cd}	0.163 ± 0.007^{c}	0.107 ± 0.005^{de}	0.534 ± 0.024^{de}	102.44 ± 4.61^{bc}	21.47 ± 0.97^{cd}
	Td	779.04 ± 35.05^{c}	1.037 ± 0.04^{g}	0.033 ± 0.001^{e}	0.172 ± 0.008^{c}	0.111 ± 0.005^{de}	0.525 ± 0.024^{de}	105.68 ± 4.75^{bc}	22.63 ± 1.02^{cd}
	Ts	728.31 ± 32.77^{d}	1.076 ± 0.04^{fg}	0.044 ± 0.002^{cd}	0.198 ± 0.009^{bc}	0.178 ± 0.008^{cd}	0.588 ± 0.026^{d}	124.32 ± 5.59^{b}	29.82 ± 1.34^{c}
	Tb	747.33 ± 33.62^{cd}	1.045 ± 0.04^{g}	0.045 ± 0.002^{c}	0.176 ± 0.008^{c}	0.174 ± 0.008^{cd}	0.646 ± 0.029^{cd}	121.84 ± 5.48^{b}	28.41 ± 1.28^{c}

TPC: Total protein content; PRO: Proline; GB: Glycine-Betaine; *Differences in the letters indicates statistical significance at 5% level in the columns.

Table S4. Antioxidant responses caused by Borax and GB applications in the stem of hulled wheats

Applications	Wheats	TPC (µg ml^{-1} protein)	SOD (U ml^{-1} protein)	CAT (U ml^{-1} protein)	GR (U ml^{-1} protein)	GST (U ml^{-1} protein)	APX (U ml^{-1} protein)	PRO (nmol gr^{-1} fw)	MDA (nmol gr^{-1} fw)
Control	Tm	271.22 ± 12.20^{fg}	0.962 ± 0.043^{g}	0.018 ± 0.001^{e}	0.117 ± 0.005^{e}	0.099 ± 0.004^{e}	0.577 ± 0.026^{e}	21.45 ± 0.97^{g}	6.52 ± 0.29^{f}
	Td	298.31 ± 13.42^{f}	1.014 ± 0.046^{f}	0.017 ± 0.001^{e}	0.117 ± 0.005^{e}	0.098 ± 0.004^{e}	0.537 ± 0.024^{ef}	24.78 ± 1.12^{g}	5.62 ± 0.25^{f}
	Ts	185.33 ± 8.34^{g}	1.144 ± 0.051^{cd}	0.034 ± 0.002^{d}	0.161 ± 0.007^{de}	0.135 ± 0.006^{de}	0.637 ± 0.029^{d}	32.05 ± 1.44^{g}	7.12 ± 0.32^{e}
	Tb	233.17 ± 10.49^{g}	1.062 ± 0.048^{e}	0.035 ± 0.002^{d}	0.146 ± 0.007^{de}	0.137 ± 0.006^{de}	0.617 ± 0.028^{de}	30.34 ± 1.37^{g}	6.74 ± 0.30^{f}
Control + 1 mM GB	Tm	641.27 ± 28.86^{ab}	1.018 ± 0.046^{f}	0.022 ± 0.001^{e}	0.124 ± 0.006^{e}	0.111 ± 0.005^{e}	0.624 ± 0.028^{d}	24.36 ± 1.10^{g}	5.44 ± 0.24^{f}
	Td	674.70 ± 30.36^{ab}	1.039 ± 0.047^{e}	0.020 ± 0.001^{e}	0.126 ± 0.006^{e}	0.109 ± 0.005^{e}	0.602 ± 0.027^{de}	30.47 ± 1.37^{g}	5.02 ± 0.23^{f}
	Ts	588.82 ± 26.50^{b}	1.205 ± 0.054^{bc}	0.039 ± 0.002^{d}	0.178 ± 0.008^{d}	0.148 ± 0.007^{d}	0.696 ± 0.031^{c}	58.36 ± 2.63^{f}	6.25 ± 0.28^{f}
	Tb	589.39 ± 26.52^{b}	1.107 ± 0.050^{d}	0.039 ± 0.002^{d}	0.165 ± 0.007^{d}	0.154 ± 0.007^{d}	0.674 ± 0.030^{cd}	57.88 ± 2.60^{f}	5.86 ± 0.26^{f}
1 mg L^{-1} Borax	Tm	361.71 ± 16.28^{e}	1.036 ± 0.047^{ef}	0.024 ± 0.001^{e}	0.134 ± 0.006^{e}	0.108 ± 0.005^{e}	0.596 ± 0.027^{de}	27.45 ± 1.24^{g}	7.08 ± 0.32^{e}
	Td	433.19 ± 19.49^{d}	1.054 ± 0.047^{e}	0.025 ± 0.001^{e}	0.132 ± 0.006^{e}	0.108 ± 0.005^{e}	0.564 ± 0.025^{e}	28.81 ± 1.30^{g}	6.96 ± 0.31^{e}
	Ts	279.29 ± 12.57^{f}	1.196 ± 0.054^{bc}	0.049 ± 0.002^{c}	0.185 ± 0.008^{d}	0.154 ± 0.007^{d}	0.663 ± 0.030^{cd}	33.56 ± 1.51^{g}	7.88 ± 0.35^{e}
	Tb	301.20 ± 13.55^{f}	1.135 ± 0.051^{cd}	0.051 ± 0.002^{c}	0.171 ± 0.008^{d}	0.159 ± 0.007^{cd}	0.655 ± 0.029^{cd}	30.24 ± 1.36^{g}	7.65 ± 0.34^{e}
1 mg L^{-1} Borax + 1 mM GB	Tm	579.03 ± 26.06^{b}	1.092 ± 0.049^{d}	0.026 ± 0.001^{de}	0.141 ± 0.006^{de}	0.124 ± 0.006^{de}	0.648 ± 0.029^{cd}	32.08 ± 1.44^{g}	5.58 ± 0.25^{f}
	Td	606.68 ± 27.30^{b}	1.104 ± 0.050^{d}	0.025 ± 0.001^{de}	0.146 ± 0.007^{de}	0.118 ± 0.005^{e}	0.625 ± 0.028^{d}	44.66 ± 2.01^{fg}	5.19 ± 0.23^{f}
	Ts	563.45 ± 25.36^{b}	1.245 ± 0.056^{ab}	0.054 ± 0.002^{c}	0.214 ± 0.010^{c}	0.172 ± 0.008^{c}	0.736 ± 0.033^{bc}	76.25 ± 3.43^{e}	6.34 ± 0.29^{f}
	Tb	557.11 ± 25.07^{bc}	1.176 ± 0.053^{c}	0.052 ± 0.002^{c}	0.203 ± 0.009^{cd}	0.185 ± 0.008^{c}	0.714 ± 0.032^{c}	72.63 ± 3.27^{e}	6.76 ± 0.30^{ef}
5 mg L^{-1} Borax	Tm	420.52 ± 18.92^{d}	1.125 ± 0.051^{cd}	0.034 ± 0.002^{d}	0.147 ± 0.007^{de}	0.124 ± 0.006^{de}	0.605 ± 0.027^{de}	51.68 ± 2.33^{f}	9.16 ± 0.41^{cd}
	Td	395.14 ± 17.78^{de}	1.129 ± 0.051^{cd}	0.038 ± 0.002^{d}	0.145 ± 0.007^{de}	0.127 ± 0.006^{de}	0.594 ± 0.027^{de}	54.76 ± 2.46^{f}	9.74 ± 0.44^{cd}
	Ts	332.89 ± 14.98^{ef}	1.246 ± 0.056^{ab}	0.063 ± 0.003^{bc}	0.206 ± 0.009^{cd}	0.215 ± 0.010^{b}	0.698 ± 0.031^{c}	72.84 ± 3.28^{e}	10.38 ± 0.47^{cd}
	Tb	366.90 ± 16.51^{e}	1.197 ± 0.054^{bc}	0.066 ± 0.003^{bc}	0.201 ± 0.009^{cd}	0.224 ± 0.010^{b}	0.702 ± 0.032^{c}	66.51 ± 2.99^{ef}	10.12 ± 0.46^{cd}
5 mg L^{-1} Borax + 1 mM GB	Tm	622.25 ± 28.00^{ab}	1.274 ± 0.057^{a}	0.044 ± 0.002^{cd}	0.193 ± 0.009^{cd}	0.144 ± 0.006^{d}	0.709 ± 0.032^{c}	76.12 ± 3.43^{e}	6.26 ± 0.28^{f}
	Td	634.36 ± 28.55^{ab}	1.193 ± 0.054^{bc}	0.043 ± 0.002^{cd}	0.204 ± 0.009^{cd}	0.142 ± 0.006^{d}	0.684 ± 0.031^{cd}	81.44 ± 3.66^{e}	6.14 ± 0.28^{f}
	Ts	674.71 ± 30.36^{a}	1.286 ± 0.058^{a}	0.075 ± 0.003^{b}	0.271 ± 0.012^{b}	0.216 ± 0.010^{b}	0.832 ± 0.037^{ab}	98.65 ± 4.44^{d}	6.78 ± 0.31^{e}
	Tb	645.31 ± 29.04^{ab}	1.243 ± 0.056^{ab}	0.077 ± 0.003^{b}	0.268 ± 0.012^{b}	0.205 ± 0.009^{bc}	0.848 ± 0.038^{ab}	103.54 ± 4.66^{d}	7.21 ± 0.32^{e}
10 mg L^{-1} Borax	Tm	444.72 ± 20.01^{d}	1.132 ± 0.051^{cd}	0.036 ± 0.002^{d}	0.151 ± 0.007^{de}	0.141 ± 0.006^{d}	0.647 ± 0.029^{cd}	84.96 ± 3.82d^{e}	14.63 ± 0.66^{c}
	Td	389.96 ± 17.55^{de}	1.139 ± 0.051^{cd}	0.038 ± 0.002^{d}	0.152 ± 0.007^{de}	0.146 ± 0.007^{d}	0.633 ± 0.028^{d}	86.52 ± 3.89^{de}	16.82 ± 0.76^{cd}
	Ts	327.71 ± 14.75^{ef}	1.254 ± 0.056^{ab}	0.065 ± 0.003^{bc}	0.225 ± 0.010^{c}	0.264 ± 0.012^{a}	0.888 ± 0.040^{ab}	114.32 ± 5.14^{cd}	15.22 ± 0.68^{cd}
	Tb	347.32 ± 15.63^{e}	1.227 ± 0.055^{b}	0.071 ± 0.003^{b}	0.233 ± 0.010^{c}	0.278 ± 0.013^{a}	0.896 ± 0.040^{a}	121.65 ± 5.47^{c}	15.78 ± 0.71^{cd}
10 mg L^{-1} Borax + 1 mM GB	Tm	720.24 ± 32.41^{a}	1.299 ± 0.058^{a}	0.047 ± 0.002^{cd}	0.208 ± 0.009^{cd}	0.157 ± 0.007^{cd}	0.749 ± 0.034^{bc}	99.11 ± 4.46^{d}	8.68 ± 0.39^{d}
	Td	762.32 ± 34.30^{a}	1.265 ± 0.057^{a}	0.047 ± 0.002^{cd}	0.219 ± 0.010^{c}	0.155 ± 0.007^{cd}	0.744 ± 0.033^{bc}	106.54 ± 4.79^{d}	8.86 ± 0.40^{d}
	Ts	786.53 ± 35.39^{a}	1.291 ± 0.058^{a}	0.088 ± 0.004^{ab}	0.322 ± 0.014^{a}	0.234 ± 0.011^{ab}	0.916 ± 0.041^{a}	113.85 ± 5.12^{cd}	8.74 ± 0.39^{d}
	Tb	744.45 ± 33.50^{a}	1.276 ± 0.057^{a}	0.094 ± 0.004^{a}	0.328 ± 0.015^{a}	0.241 ± 0.011^{ab}	0.945 ± 0.043^{a}	122.56 ± 5.52^{c}	9.08 ± 0.41^{d}
15 mg L^{-1} Borax	Tm	435.49 ± 19.60^{d}	1.013 ± 0.046^{f}	0.033 ± 0.001^{d}	0.134 ± 0.006^{e}	0.123 ± 0.006^{de}	0.603 ± 0.027^{de}	86.22 ± 3.88^{de}	27.32 ± 1.23^{bc}
	Td	364.60 ± 16.41^{e}	1.016 ± 0.046^{f}	0.034 ± 0.002^{d}	0.135 ± 0.006^{e}	0.125 ± 0.006^{de}	0.584 ± 0.026^{de}	91.38 ± 4.11^{de}	31.63 ± 1.42^{b}
	Ts	275.83 ± 12.41^{f}	1.066 ± 0.048^{de}	0.051 ± 0.002^{c}	0.176 ± 0.008^{d}	0.212 ± 0.010^{b}	0.645 ± 0.029^{cd}	124.68 ± 5.61^{c}	32.55 ± 1.46^{b}
	Tb	309.26 ± 13.92^{f}	1.044 ± 0.047^{e}	0.054 ± 0.002^{c}	0.182 ± 0.008^{d}	0.216 ± 0.010^{b}	0.696 ± 0.031^{c}	146.57 ± 6.60^{b}	30.87 ± 1.39^{b}
15 mg L^{-1} Borax + 1 mM GB	Tm	862.25 ± 38.80^{a}	1.232 ± 0.055^{b}	0.043 ± 0.002^{cd}	0.171 ± 0.008^{d}	0.138 ± 0.006^{d}	0.684 ± 0.031^{cd}	102.98 ± 4.63^{d}	12.22 ± 0.55^{c}
	Td	864.92 ± 38.92^{a}	1.210 ± 0.054^{bc}	0.042 ± 0.002^{cd}	0.176 ± 0.008^{d}	0.136 ± 0.006^{de}	0.678 ± 0.031^{cd}	109.36 ± 4.92^{cd}	12.38 ± 0.56^{c}
	Ts	879.33 ± 39.57^{a}	1.225 ± 0.055^{b}	0.063 ± 0.003^{bc}	0.228 ± 0.010^{c}	0.163 ± 0.007^{cd}	0.751 ± 0.034^{bc}	146.54 ± 6.59^{b}	11.65 ± 0.52^{c}
	Tb	813.38 ± 36.60^{a}	1.211 ± 0.054^{bc}	0.069 ± 0.003^{b}	0.227 ± 0.010^{c}	0.168 ± 0.008^{cd}	0.779 ± 0.035^{b}	163.08 ± 7.34^{ab}	11.24 ± 0.51^{c}
20 mg L^{-1} Borax	Tm	310.42 ± 13.97^{f}	0.928 ± 0.042^{g}	0.030 ± 0.001^{de}	0.128 ± 0.006^{e}	0.112 ± 0.005^{e}	0.553 ± 0.025^{e}	108.78 ± 4.90^{d}	63.54 ± 2.86^{ab}
	Td	294.85 ± 13.27^{f}	0.926 ± 0.042^{g}	0.029 ± 0.001^{de}	0.130 ± 0.006^{e}	0.115 ± 0.005^{e}	0.509 ± 0.023^{f}	110.21 ± 4.96^{cd}	68.79 ± 3.10^{ab}
	Ts	232.03 ± 10.44^{g}	0.954 ± 0.043^{g}	0.038 ± 0.002^{d}	0.157 ± 0.007^{de}	0.155 ± 0.007^{cd}	0.579 ± 0.026^{e}	135.64 ± 6.10^{bc}	75.03 ± 3.38^{a}
	Tb	236.63 ± 10.65^{g}	0.941 ± 0.042^{g}	0.039 ± 0.002^{d}	0.160 ± 0.007^{de}	0.158 ± 0.007^{cd}	0.594 ± 0.027^{de}	151.38 ± 6.81^{b}	73.28 ± 3.30^{a}
20 mg L^{-1} Borax + 1 mM GB	Tm	708.71 ± 31.89^{a}	1.077 ± 0.048^{de}	0.038 ± 0.002^{d}	0.154 ± 0.007^{de}	0.111 ± 0.005^{e}	0.502 ± 0.023^{f}	128.36 ± 5.78^{c}	26.30 ± 1.18^{bc}
	Td	727.16 ± 32.72^{a}	1.063 ± 0.048^{e}	0.036 ± 0.002^{d}	0.155 ± 0.007^{de}	0.108 ± 0.005^{e}	0.496 ± 0.022^{f}	121.47 ± 5.47^{c}	28.65 ± 1.29^{bc}
	Ts	743.87 ± 33.47^{a}	1.088 ± 0.049^{de}	0.039 ± 0.002^{d}	0.176 ± 0.008^{d}	0.122 ± 0.005^{de}	0.534 ± 0.024^{ef}	186.69 ± 8.40^{a}	27.82 ± 1.25^{bc}
	Tb	751.36 ± 33.81^{a}	1.081 ± 0.049^{de}	0.041 ± 0.002^{cd}	0.179 ± 0.008^{d}	0.124 ± 0.006^{de}	0.547 ± 0.025^{e}	194.02 ± 8.73^{a}	24.81 ± 1.12^{bc}

TPC: Total protein content; PRO: Proline; GB: Glycine-Betaine; *Differences in the letters indicates statistical significance at 5% level in the columns.

Table S5. Tests of Between-Subjects Effects

Source	Dependent Variable	Type III Sum of Squares	df	Mean Square	F	P
Corrected Model	ProtCons	2116261.80	95	22276.44	161.20	0.000
	SOD	5.03	95	0.05	9.15	0.000
	CAT	0.09	95	0.01	1.33	0.000
	GR	1.14	95	0.01	11.66	0.000
	GST	0.40	95	0.01	1.19	0.000
	APX	0.52	95	0.01	41.69	0.000
	Prolin	10089516.04	95	106205.43	646.24	0.000
	MDA	4404563.47	95	46363.82	326.63	0.000
Wheats	ProtCons	65844.20	3	21948.07	247.35	0.000
	SOD	1.35	3	0.45	124.38	0.000
	CAT	0.02	3	0.01	14.01	0.000
	GR	0.47	3	0.16	242.72	0.000
	GST	0.06	3	0.02	10.17	0.000
	APX	0.09	3	0.03	353.19	0.000
	Prolin	188303.01	3	62767.66	621.14	0.000
	MDA	61835.92	3	20611.97	220.96	0.000
Sections	ProtCons	201488.03	1	201488.03	2270.74	0.000
	SOD	0.99	1	0.99	273.46	0.000
	CAT	0.01	1	0.01	25.18	0.000
	GR	0.10	1	0.10	158.71	0.000
	GST	0.01	1	0.01	4.25	0.010
	APX	0.01	1	0.01	120.27	0.000
	Prolin	77351.10	1	77351.10	765.46	0.000
	MDA	2478.10	1	2478.10	26.56	0.000
Doses	ProtCons	1636285.28	11	148753.20	1024.48	0.000
	SOD	1.86	11	0.17	28.44	0.000
	CAT	0.01	11	0.01	2.10	0.000
	GR	0.39	11	0.04	33.84	0.000
	GST	0.13	11	0.01	3.24	0.000
	APX	0.32	11	0.03	215.13	0.000
	Prolin	9305801.88	11	845981.99	5116.11	0.000
	MDA	4446470.90	11	404224.62	2648.19	0.000
Wheats x Sections	ProtCons	355.01	3	118.33	1.33	**0.106**
	SOD	0.01	3	0.01	0.85	**0.175**
	CAT	0.01	3	0.01	2.82	0.007
	GR	0.04	3	0.01	19.49	0.000
	GST	0.01	3	0.01	0.99	**0.205**
	APX	0.01	3	0.01	5.30	0.000
	Prolin	2182.79	3	727.59	7.20	0.000
	MDA	950.74	3	316.91	3.39	0.001
Wheats x Doses	ProtCons	45245.48	33	1371.07	9.44	0.000
	SOD	0.36	33	0.01	1.85	0.000
	CAT	0.02	33	0.01	0.76	**0.179**
	GR	0.08	33	0.01	2.29	0.000
	GST	0.08	33	0.01	0.69	**0.268**
	APX	0.02	33	0.01	3.32	0.000
	Prolin	53372.47	33	1617.34	9.78	0.000
	MDA	36460.51	33	1104.86	7.23	0.000
Sections x Doses	ProtCons	202735.30	11	18430.48	126.93	0.000
	SOD	0.22	11	0.02	3.33	0.000
	CAT	0.01	11	0.01	0.65	**0.334**
	GR	0.03	11	0.01	2.10	0.000
	GST	0.02	11	0.01	0.59	**0.501**
	APX	0.08	11	0.01	51.61	0.000
	Prolin	191519.74	11	17410.88	105.29	0.000
	MDA	32277.73	11	2934.33	19.22	0.000
Wheats x Sections x Doses	ProtCons	12388.54	33	375.41	2.58	0.000
	SOD	0.22	33	0.01	1.11	0.007
	CAT	0.02	33	0.01	0.65	**0.449**
	GR	0.03	33	0.01	0.78	**0.139**
	GST	0.08	33	0.01	0.66	**0.311**
	APX	0.01	33	0.01	2.31	0.000
	Prolin	39712.87	33	1203.42	7.27	0.000
	MDA	13549.12	33	410.58	2.69	0.000

a. R Squared = 0.990 (Adjusted R Squared = 0.989); b. R Squared = 0.877 (Adjusted R Squared = 0.820); c. R Squared = 0.515 (Adjusted R Squared = 0.270); d. R Squared = 0.901 (Adjusted R Squared = 0.856); e. R Squared = 0.488 (Adjusted R Squared = 0.229); f. R Squared = 0.970 (Adjusted R Squared = 0.955); g. R Squared = 0.997 (Adjusted R Squared = 0.998); h. R Squared = 0.997 (Adjusted R Squared = 0.995)

Table S6. Levene's Test of Equality of Error Variances[a]

		Levene Statistic	df1	df2	P
ProtCons	Based on Mean	2.201	95	190.629	0.001
	Based on Median	0.559	95	190.629	0.761
	Based on Median and with adjusted df	0.559	95	26.030	0.716
	Based on trimmed mean	2.030	95	190.629	0.001
SOD	Based on Mean	9.406	95	190.629	0.001
	Based on Median	0.627	95	190.629	0.548
	Based on Median and with adjusted df	0.627	95	2.449	0.561
	Based on trimmed mean	7.483	95	190.629	0.001
CAT	Based on Mean	9.806	95	190.629	0.001
	Based on Median	0.622	95	190.629	0.505
	Based on Median and with adjusted df	0.622	95	2.484	0.582
	Based on trimmed mean	7.740	95	190.629	0.001
GR	Based on Mean	9.584	95	190.629	0.001
	Based on Median	0.625	95	190.629	0.529
	Based on Median and with adjusted df	0.625	95	1.356	0.662
	Based on trimmed mean	7.601	95	190.629	0.001
GST	Based on Mean	9.910	95	190.629	0.001
	Based on Median	0.629	95	190.629	0.448
	Based on Median and with adjusted df	0.629	95	1.263	0.602
	Based on trimmed mean	7.821	95	190.629	0.001
APX	Based on Mean	6.790	95	190.629	0.001
	Based on Median	0.586	95	190.629	0.656
	Based on Median and with adjusted df	0.586	95	4.578	0.649
	Based on trimmed mean	5.629	95	190.629	0.001
Prolin	Based on Mean	7.036	95	190.629	0.001
	Based on Median	0.560	95	190.629	0.79
	Based on Median and with adjusted df	0.560	95	6.105	0.652
	Based on trimmed mean	5.768	95	190.629	0.001
MDA	Based on Mean	9.100	95	190.629	0.001
	Based on Median	0.612	95	190.629	0.518
	Based on Median and with adjusted df	0.612	95	1.314	0.621
	Based on trimmed mean	7.263	95	190.629	0.001
Tests the null hypothesis that the error variance of the dependent variable is equal across groups.					
a. Design: Intercept + Wheats + Sections + Doses + Wheats x Sections + Wheats x Doses + Sections x Doses + Wheats x Sections x Doses					

Table S7. Multiple comparisons of hulled wheat species

Dependent Variable		(I) Wheat	(J) Wheat	Mean Difference (I-J)	Std. Error	P	95% Confidence Interval Lower Bound	95% Confidence Interval Upper Bound
ProtCons	LSD	*T. monococcum*	*T. dicoccum*	-3.454	78.519	0.000	-49.998	-19.096
			T. speltoides	17.693	78.519	0.000	161.480	192.383
			T. boeoticum	16.622	78.519	0.000	150.774	181.676
		T. dicoccum	*T. monococcum*	3.45	78.519	0.000	19.096	49.998
			T. speltoides	21.147	78.519	0.000	196.028	226.930
			T. boeoticum	20.077	78.519	0.000	185.322	216.224
		T. speltoides	*T. monococcum*	-17.693	78.519	0.000	-192.383	-161.480
			T. dicoccum	-21.147	78.519	0.000	-226.930	-196.028
			T. boeoticum	-10.706	78.519	**0.196**	-26.157	0.474
		T. boeoticum	*T. monococcum*	-16.622	78.519	0.000	-181.676	-150.774
			T. dicoccum	-20.077	78.519	0.000	-216.224	-185.322
			T. speltoides	10.706	78.519	**0.196**	-0.474	26.157
SOD	LSD	*T. monococcum*	*T. dicoccum*	0.015	0.005	0.003	0.006	0.026
			T. speltoides	-0.089	0.005	0.000	-0.100	-0.080
			T. boeoticum	-0.061	0.005	0.000	-0.072	-0.052
		T. dicoccum	*T. monococcum*	-0.015	0.005	0.003	-0.026	-0.006
			T. speltoides	-0.105	0.005	0.000	-0.115	-0.096
			T. boeoticum	-0.077	0.005	0.000	-0.087	-0.068
		T. speltoides	*T. monococcum*	0.089	0.005	0.000	0.080	0.100
			T. dicoccum	0.105	0.005	0.000	0.096	0.115
			T. boeoticum	0.027	0.005	0.000	0.018	0.038
		T. boeoticum	*T. monococcum*	0.061	0.005	0.000	0.052	0.072
			T. dicoccum	0.077	0.005	0.000	0.068	0.087
			T. speltoides	-0.027	0.005	0.000	-0.038	-0.018
CAT	LSD	*T. monococcum*	*T. dicoccum*	0.001	0.002	**0.651**	-0.004	0.004
			T. speltoides	-0.010	0.002	0.000	-0.014	-0.007
			T. boeoticum	-0.010	0.002	0.000	-0.014	-0.007
		T. dicoccum	*T. monococcum*	0.001	0.002	**0.651**	-0.004	0.004
			T. speltoides	-0.010	0.002	0.000	-0.014	-0.007
			T. boeoticum	-0.010	0.002	0.000	-0.014	-0.007
		T. speltoides	*T. monococcum*	0.010	0.002	0.000	0.007	0.014
			T. dicoccum	0.010	0.002	0.000	0.007	0.014
			T. boeoticum	0.001	0.002	**0.412**	-0.003	0.004
		T. boeoticum	*T. monococcum*	0.010	0.002	0.000	0.007	0.014
			T. dicoccum	0.010	0.002	0.000	0.007	0.014
			T. speltoides	-0.001	0.002	**0.412**	-0.004	0.003
GR	LSD	*T. monococcum*	*T. dicoccum*	0.001	0.002	**0.279**	-0.003	0.005
			T. speltoides	-0.055	0.002	0.000	-0.059	-0.051
			T. boeoticum	-0.044	0.002	0.000	-0.049	-0.041
		T. dicoccum	*T. monococcum*	-0.001	0.002	**0.279**	-0.005	0.003
			T. speltoides	-0.056	0.002	0.000	-0.060	-0.052
			T. boeoticum	-0.045	0.002	0.000	-0.050	-0.042
		T. speltoides	*T. monococcum*	0.055	0.002	0.000	0.051	0.059
			T. dicoccum	0.056	0.002	0.000	0.052	0.060
			T. boeoticum	0.010	0.002	0.000	0.006	0.014
		T. boeoticum	*T. monococcum*	0.044	0.002	0.000	0.041	0.049
			T. dicoccum	0.045	0.002	0.000	0.042	0.050
			T. speltoides	-0.010	0.002	0.000	-0.014	-0.006
GST	LSD	*T. monococcum*	*T. dicoccum*	-0.011	0.004	0.007	-0.020	-0.004
			T. speltoides	-0.027	0.004	0.000	-0.035	-0.019
			T. boeoticum	-0.015	0.004	0.000	-0.023	-0.007
		T. dicoccum	*T. monococcum*	0.011	0.004	0.007	0.004	0.020
			T. speltoides	-0.015	0.004	0.000	-0.023	-0.008
			T. boeoticum	-0.003	0.004	**0.104**	-0.011	0.004
		T. speltoides	*T. monococcum*	0.027	0.004	0.000	0.019	0.035
			T. dicoccum	0.015	0.004	0.000	0.008	0.023
			T. boeoticum	0.011	0.004	0.006	0.004	0.020
		T. boeoticum	*T. monococcum*	0.015	0.004	0.000	0.007	0.023
			T. dicoccum	0.003	0.004	**0.104**	-0.004	0.011
			T. speltoides	-0.011	0.004	0.006	-0.020	-0.004
APX	LSD	*T. monococcum*	*T. dicoccum*	0.013	0.001	0.000	0.012	0.015
			T. speltoides	-0.017	0.001	0.000	-0.019	-0.016
			T. boeoticum	-0.007	0.001	0.000	-0.010	-0.007

		T. dicoccum	*T. monococcum*	-0.013	0.001	0.000	-0.015	-0.012
			T. speltoides	-0.030	0.001	0.000	-0.032	-0.029
			T. boeoticum	-0.021	0.001	0.000	-0.023	-0.020
		T. speltoides	*T. monococcum*	0.017	0.001	0.000	0.016	0.019
			T. dicoccum	0.030	0.001	0.000	0.029	0.032
			T. boeoticum	0.009	0.001	0.000	0.008	0.011
		T. boeoticum	*T. monococcum*	0.007	0.001	0.000	0.007	0.010
			T. dicoccum	0.021	0.001	0.000	0.020	0.023
			T. speltoides	-0.009	0.001	0.000	-0.011	-0.008
Proline	LSD	*T. monococcum*	*T. dicoccum*	-18.976	83.642	0.000	-206.227	-173.309
			T. speltoides	-42.784	83.642	0.000	-444.307	-411.389
			T. boeoticum	-33.320	83.642	0.000	-349.660	-316.742
		T. dicoccum	*T. monococcum*	18.976	83.642	0.000	173.309	206.227
			T. speltoides	-23.807	83.642	0.000	-254.538	-221.621
			T. boeoticum	-14.343	83.642	0.000	-159.892	-126.973
		T. speltoides	*T. monococcum*	42.784	83.642	0.000	411.389	444.307
			T. dicoccum	23.807	83.642	0.000	221.621	254.538
			T. boeoticum	9.464	83.642	0.000	78.188	111.106
		T. boeoticum	*T. monococcum*	33.320	83.642	0.000	316.742	349.660
			T. dicoccum	14.343	83.642	0.000	126.973	159.892
			T. speltoides	-9.464	83.642	0.000	-111.106	-78.188
MDA	LSD	*T. monococcum*	*T. dicoccum*	5.256	80.357	0.000	36.754	68.379
			T. speltoides	-17.710	80.357	0.000	-192.915	-161.290
			T. boeoticum	-12.506	80.357	0.000	-140.873	-109.248
		T. dicoccum	*T. monococcum*	-5.256	80.357	0.000	-68.379	-36.754
			T. speltoides	-22.966	80.357	0.000	-245.481	-213.856
			T. boeoticum	-17.762	80.357	0.000	-193.440	-161.815
		T. speltoides	*T. monococcum*	17.710	80.357	0.000	161.290	192.915
			T. dicoccum	22.966	80.357	0.000	213.856	245.481
			T. boeoticum	5.204	80.357	0.000	36.230	67.855
		T. boeoticum	*T. monococcum*	12.506	80.357	0.000	109.248	140.873
			T. dicoccum	17.762	80.357	0.000	161.815	193.440
			T. speltoides	-5.204	80.357	0.000	-67.855	-36.230

Based on observed means. The error term is Mean Square(Error) = 92.988. * The mean difference is significant at the 0.05 level.

Table S.8 MANOVA-Multivariate Tests[a]

Effect		Value	F	Hypothesis df	Error df	P
Intercept	Pillai's Trace	0.629	219221.996	8	186.225	0.000
	Wilks' Lambda	0.000	219221.996	8	186.225	0.000
	Hotelling's Trace	5924.919	219221.996	8	186.225	0.000
	Roy's Largest Root	5924.919	219221.996	8	186.225	0.000
Wheats	Pillai's Trace	1.187	39.803	24	562.450	0.000
	Wilks' Lambda	0.005	96.164	24	540.487	0.000
	Hotelling's Trace	17.153	210.608	24	556.158	0.000
	Roy's Largest Root	15.058	560.929	8	187.483	0.000
Sections	Pillai's Trace	0.597	434.542	8	186.225	0.000
	Wilks' Lambda	0.032	434.542	8	186.225	0.000
	Hotelling's Trace	11.744	434.542	8	186.225	0.000
	Roy's Largest Root	11.744	434.542	8	186.225	0.000
Doses	Pillai's Trace	3.021	15.900	88	1525033.113	0.000
	Wilks' Lambda	0.000	110.197	88	1378685.298	0.000
	Hotelling's Trace	552.429	1128.835	88	1480993.377	0.000
	Roy's Largest Root	380.253	6400.932	11	190629.139	0.000
Wheat x Sections	Pillai's Trace	0.2950	4.345	24	562.450	0.000
	Wilks' Lambda	0.360	4.761	24	540.487	0.000
	Hotelling's Trace	0.422	5.185	24	556.158	0.000
	Roy's Largest Root	0.343	12.784	8	187.483	0.000
Wheat x Doses	Pillai's Trace	2.232	2.815	264	1525.033	0.000
	Wilks' Lambda	0.002	3.503	264	1486.669	0.000
	Hotelling's Trace	6.410	4.366	264	1480.993	0.000
	Roy's Largest Root	2.325	13.051	33	190.629	0.000
Sections x Doses	Pillai's Trace	2.302	8.932	88	1525.033	0.000
	Wilks' Lambda	0.000	19.109	88	1378.685	0.000
	Hotelling's Trace	18.752	38.317	88	1480.993	0.000
	Roy's Largest Root	9.928	167.127	11	190.629	0.000
Wheats x Sections x Doses	Pillai's Trace	1.614	1.667	264	1525.033	0.000
	Wilks' Lambda	0.018	1.912	264	1486.669	0.000
	Hotelling's Trace	3.294	2.244	264	1480.993	0.000
	Roy's Largest Root	1.429	8.019	33	190.629	0.000

a. Design: Intercept + Wheats + Sections + Doses + Wheats x Sections + Wheats x Doses + Sections x Doses + Wheats x Sections x Doses
